# Student peer mentoring: Feasibility and acceptability of mHealth-based tool for alcohol and substance abuse prevention by peer mentors at a university in Kenya

**DOI:** 10.1371/journal.pdig.0000177

**Published:** 2023-01-12

**Authors:** Catherine Mawia Musyoka, Anne Mbwayo, Dennis M. Donovan, Muthoni Mathai

**Affiliations:** 1 Department of Psychiatry, College of Health Sciences, University of Nairobi, Nairobi, Kenya; 2 Department of Psychiatry & Behavioural Sciences and Alcohol & Drug Abuse Institute, University of Washington School of Medicine, Seattle, Washington State United States of America; University of Bayreuth: Universitat Bayreuth, GERMANY

## Abstract

**Objective:**

The use of mobile health (mHealth)-based interventions for the prevention of alcohol and other psychoactive substances use is an emerging practice for which new evidence is required. This study evaluated the feasibility and acceptability of a mHealth-based peer mentoring tool for early screening, brief intervention, and referral of students who abuse alcohol and other psychoactive substances. It compared the implementation of a mHealth-delivered intervention to the paper-based practice that is the standard at the University of Nairobi.

**Methods:**

A quasi-experimental study using purposive sampling was used to select a cohort of n = 100 (51 experimental, 49 control) first-year student peer mentors on two campuses of the University of Nairobi in Kenya. Data were collected on the mentors’ sociodemographic characteristics as well as the feasibility and acceptability of the interventions by way of, the magnitude of reach, feedback to investigators, referral of cases, and perceived ease of use.

**Results:**

The mHealth-based peer mentoring tool scored high with 100% of users rating it as feasible and acceptable. Among the two study cohorts, there were no differences in the acceptability of the peer mentoring intervention. When comparing the feasibility of the peer mentoring practice, actual use of the interventions, and intervention reach, the mHealth-based cohort mentored four mentees for every one mentored by the standard practice cohort.

**Conclusion:**

The mHealth-based peer mentoring tool had high feasibility and acceptability among student peer mentors. The intervention provided evidence for the need to expand the availability of screening services for alcohol and other psychoactive substances use among students in the university and promote the appropriate management practices within and outside the university.

## Author summary

Given the documented prevalence of harmful alcohol and other psychoactive substances use among young adults, the need for innovative ways to communicate behaviour change is urgent. The harmful use of psychoactive substances has a myriad of negative consequences for young adults both in the short term and long term. These eventually lead to poor life outcomes including loss of lives. Currently, there is a need for more evidence on the feasibility and acceptability of mHealth-based interventions for the prevention of the harmful use of psychoactive substances among young adults. This study adds to the body of evidence about efficacious measures for the prevention of the harmful use of alcohol and other substances among young adults. It is relevant in providing data on the feasibility and acceptability of mHealth-based peer mentoring in a university setting. Given the limited evidence of the effectiveness of mHealth-based interventions in Africa, this study adds evidence to this important but emerging practice. Finally, this study contributes to the achievement of the Global Sustainable Development Goals, specifically strengthening the prevention and treatment of substance abuse, including narcotic drug abuse and harmful use of alcohol.

## Introduction

Excessive consumption of alcohol and other psychoactive substances among the young adult population is an issue of global health concern [1]. In Kenya, alcohol and substance use among college students is also a growing public health problem [2]. The prevalence of alcohol and substance use among students in Kenya is high, with ranges between 20–67% [3–5]. Although alcohol is the most used substance by Kenyan university students, there is a rising trend in cannabis and tobacco use. Also, there is an emerging increase in opioid and sedative use among university students [3]. When compared across the sexes, male students have a higher prevalence of harmful alcohol and substance use than female students [3,5,6].

The harmful use of alcohol and other psychoactive substances among Kenyan students has been associated with widespread indiscipline and violence at school including the burning of schools and the destruction of school property [7]. Furthermore, the harmful use of alcohol and other psychoactive substances among university students poses both short-term and long-term harm including missed classes and poor academic performance [8]. Other problems include accidental injuries as a result of intoxication, criminal behaviour sexual assault, risky sexual behaviour leading to sexually transmitted infections, and unplanned pregnancies [9,10]. Since harmful alcohol and psychoactive substance use by college students negatively affects their overall life outcomes, it is important to prioritize harmful alcohol and psychoactive substance prevention interventions among university students.

Institutions of higher learning use various programs for the prevention of harmful alcohol and other psychoactive substance use; most of these programs focus on individual students, educating them on the negative effects of substance use [11].

These programs include harmful psychoactive substance sensitization forums as well as awareness creation on the rules and regulations regarding alcohol and psychoactive substance use in the institutions of higher learning.

The National Institute of Health has developed the ‘collegeAIM matrix’ which outlines over 60 college students’ substance use prevention interventions. These interventions include a mix of individual and environmental strategies that universities can customize for the prevention of alcohol and substance use among their students. The individual-level strategies are the use of college media to educate students about the risks of excessive alcohol drinking and the use of other psychoactive substances both licit and illicit. While the environmental-level strategies include enforcement of laws on underage drinking, use of sanctions for violation of drinking, and substance use laws [12]. There are, however, challenges with implementing drug prevention interventions among students. Students are a demographic group that is not very open to social information from ‘authority figures’ and this is compounded by the fact that alcohol and substance use in institutions of higher learning has overtones of a rite of passage; thus only a small number of college students seek professional support for alcohol abuse-related problems [13]. This, therefore, calls for novel and targeted prevention strategies aimed at preventing college students from initiating substance use behaviours [14].

The use of peer mentoring to prevent harmful substance use among young adults is an emerging practice in many institutions of learning. Mentoring is a structured and trusting relationship that brings people together to interact on activities of shared interests. Peer mentoring has been defined broadly as the practice of involving persons who share the same characteristics like age, sex, or same background to bring about a positive change [15].

The Big Brothers Big Sisters (BBBS) study in the USA reported a successful large-scale mentoring program that showed evidence of effectiveness and success in mentoring to reduce substance use among adolescents [16]. Peer mentoring enhances communication among young adults and breaks the power imbalance often experienced when older persons attempt to communicate behaviour change to young adults. Young adults are more likely to listen to and act on information presented by their peers as they have shared experiences.

Compared to non-peer mentors, peer mentors have higher credibility among their peers and interact with each other in a non-threatening manner, thus their messages are better received.

This is because these are young adults with whom they identify and respect and from whom they model behaviour [15]. A systematic review of peer mentoring programs reported that they are effective for both promotive and preventive interventions for substance use among young adults [17]. Moreover, peer mentoring has been used in successful behaviour change interventions to encourage young adults to embrace active physical activities [18] and to prevent substance use [19].

Mobile Health (mHealth) refers to the use of mobile phones and wireless technologies to support the achievement of health objectives, which includes interventions, research, and for healthcare delivery in public health practice [20]. The use of mHealth-based interventions for the prevention of harmful alcohol and other psychoactive substance use is an emerging field of research interest for many professionals. There is growing evidence in developed countries on the significance of the use of mHealth-based interventions for healthcare practices and substance use management [21]. However, there is little evidence to inform mHealth-based interventions’ usability and effectiveness, especially in Africa [22].

A scoping review was done in Sub-Saharan Africa (SSA) countries on the use of mHealth-based interventions to support and improve healthcare delivery.

The findings indicated that, although some progress has been realized in the implementation of mHealth interventions, there are still gaps in the evidence of their effect on the quality of service delivery which should be fully explored [23].

A systematic review of the literature provided evidence that the use of mHealth, particularly text messaging, was an acceptable, affordable and effective way to deliver messages about reducing alcohol consumption to the young adult population [20]. Another systemic review on the efficacy of mHealth interventions among young adults highlighted the advantage of the ease and convenience of the interventions; further, the majority of studies provided support that mHealth interventions were efficacious in reducing substance use [24].

mHealth-based interventions for alcohol and substance use disorder (SUD) among college students present opportunities to reach them via their preferred social media forums like WhatsApp, Instagram, TikTok, and Facebook [24]. These forums also confer anonymity to social interactions among young adults which further increases their appeal. mHealth-based strategies also have been reported to include other advantages such as the potential to play a crucial role in early diagnosis and provision of continuing care for students with alcohol and SUD [24,25].

The development of technological-based models that facilitate the early detection, diagnosis, and of effective behavioural therapies targeting alcohol and substance use disorders (SUDs) is a research priority [28]. The current face-to-face models of delivering these intervention strategies have faced challenges as they require the physical meeting of the young adult and a counsellor; they are also time-consuming and expensive; which makes them difficult to implement among college students. Furthermore, the stigma associated with alcohol and SUD contributes to only 10–15% of the affected young adults receiving the needed interventions [14].

Innovative interventions, based on newer models, that can reach more college students while maximizing the scarce resources available for the provision of SUDs prevention services are therefore needed and important to prioritize.

However, it is necessary to gain more knowledge and evidence about their acceptability and effectiveness from the peer mentors’ point of view.

The research question addressed by this study was: does the use of a mHealth-based decision support tool for peer mentoring improve reach, information provision, and treatment referral of students with harmful alcohol and other substance use problems in a university setting? While the objective of the study was to determine the feasibility and acceptability of a mHealth-based tool for peer mentoring in the prevention of alcohol and substance use among students in a university setting.

## Materials and methods

### Study setting

This study was conducted among first-year university student peer mentors on two purposively sampled campuses out of the ten campuses of the University of Nairobi, Kenya. One campus has the College of Biological and Physical Sciences which teaches science-based programs. This campus was selected to implement the mHealth-based intervention.

The second campus housed the College of Education and External Studies which teaches pedagogy-based courses and was selected to implement the standard practice of paper and pen intervention. The courses offered on these campuses were all 4-year degree programs. The selection of these study campuses was based on the documented high prevalence rates of harmful alcohol and psychoactive substance use among the students of both campuses [6]. It was, therefore, presumed that the need for psychoactive substance prevention and intervention services was higher than on other campuses.

### Study design

The study adopted a quasi-experimental design to implement the mHealth-based peer mentoring program for psychoactive substance use screening, brief intervention, and referral as compared to the standard practice intervention which used a paper and pen guide (S1 File).

The standard practice intervention collected the mentees’ biodata, followed by alcohol and substance use screening which used AUDIT and ASSIST tools. Then an appropriate information provision exercise followed, based on the identified problems.

### The mHealth app

The experimental mHealth intervention cohort used a mHealth app that was developed by the principal researcher using the Open Data Kit (ODK) technology. ODK is open-source software that has been used to design and develop android-based mobile health applications. The software can implement skip logic, validation checks, and algorithm-based decision support [25,26]. To design the mHealth app, standard psychometric tests were used.

The Alcohol Use Disorders Identification Test (AUDIT) and the Alcohol, Smoking, and Substance Involvement Screening Test (ASSIST V3.0) were programmed into ODK.

Inbuilt algorithms were programmed to calculate the subject’s specific substance use scores; these scores then determined the pathway for brief intervention and/or referral and linkage to care and support by the mHealth app.

**Fig 1:**
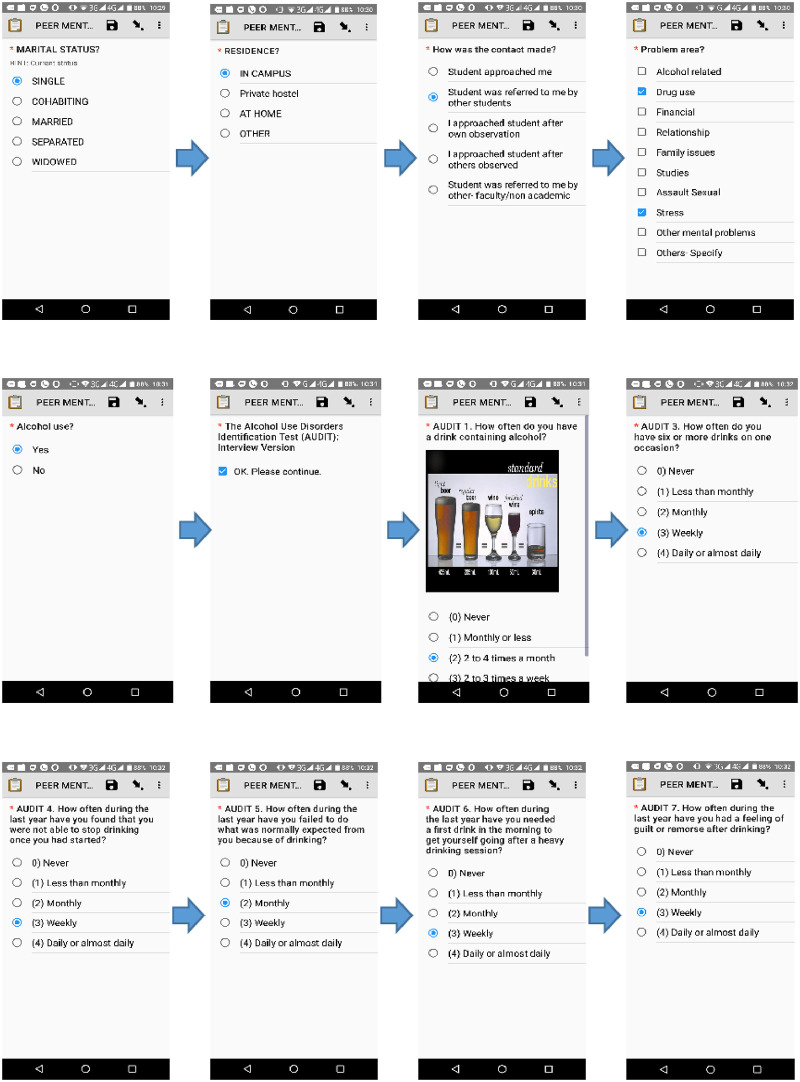
Screen-by-screen access to the mHealth-based tool.

Screen-by-screen questions based on AUDIT and ASSIST were incorporated into the mHealth app; which was then downloaded to the peer mentors’ smartphones. The process of responding to these questions was administered by the peer mentors. Fig 1 shows the screen-by-screen access of the mHealth app. To enhance confidentially and ensure that all student mentees gave honest answers, no identifiers were used; participants’ study identifiers were generated using an inbuilt algorithm.

**Fig 2:**
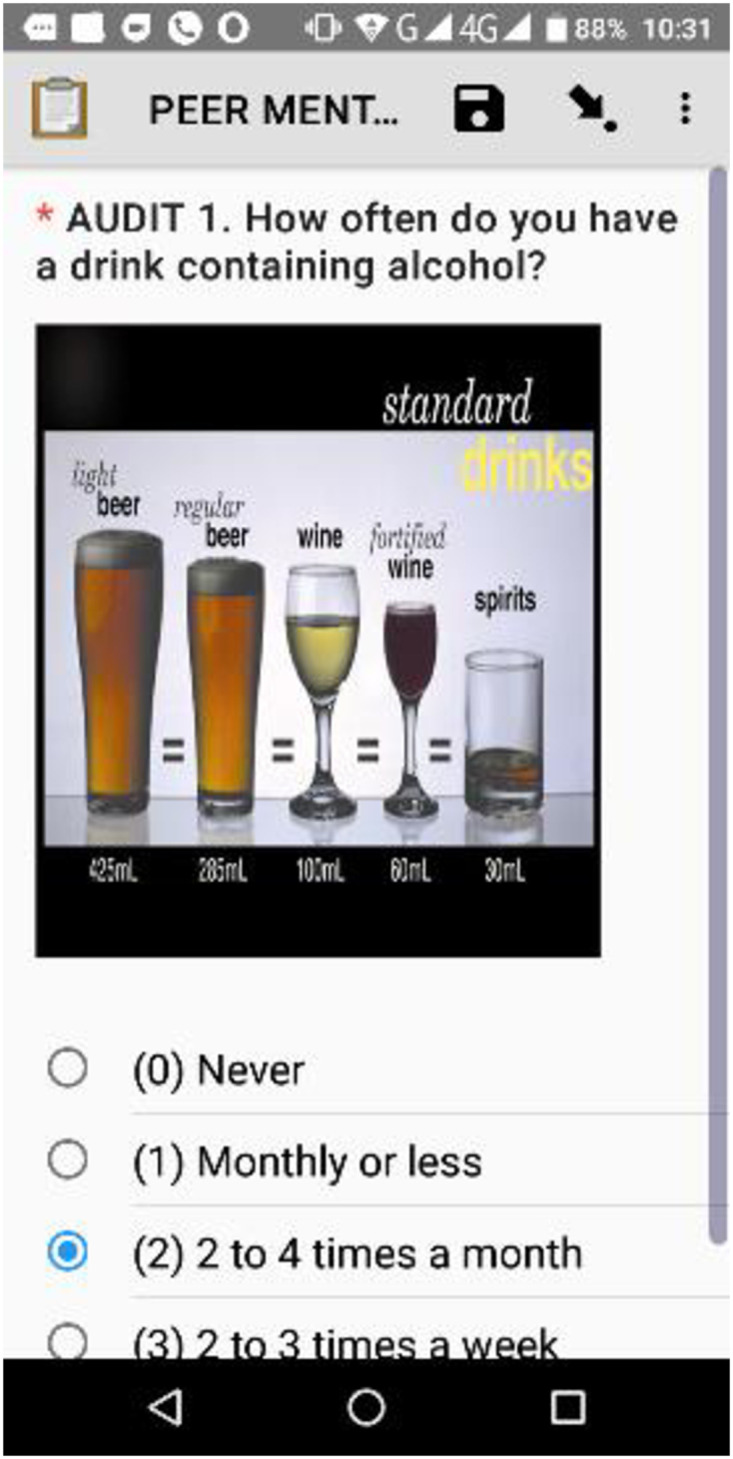
A screen image on the mHealth-based tool.

Images were also inserted in the mHealth app to enable mentees to better understand questions and be able to provide correct answers. Fig 2 is an example of an image that was inbuilt into the mobile app to assist the mentee to quantify their alcohol intake.

### Participant characteristics

The participants were undergraduate male and female student peer mentors aged between 18 years to 25 years who were transitioning from high school to either of the two campuses for their college education. The selection was based on laid down criteria that the university uses to recruit peer mentors every year. The students were chosen as peer mentors in their first year of study, and they scored a B+ or above in the Kenya certificate of secondary examination. Moreover, they demonstrated involvement in school clubs and leadership skills.

They had indicated their interest in the peer mentoring program, being role models to their fellow students, and informing them about the negative consequences of alcohol and substance use.

### Study ethics approval and informed consent

The protocol was reviewed and approved by the Kenyatta National Hospital and the University of Nairobi Ethical Committee (KNH-UoN ERC) P98/02/2018 and licensed by NACOSTI, No: NACOSTI/P/20/5582. Standard ethical research practices were maintained throughout this study. Explanations were done to all study participants and their informed written consent was obtained. Those students who opted not to take part in this study suffered no discrimination as they continued to receive all the normal university Alcohol and Drug Abuse prevention program services. Those who chose to participate in the study received no compensation. However, the participants using the mHealth-based intervention were compensated with an equivalent of 2 dollars per month for data bundles that were used for uploading the completed intervention forms.

### Inclusion and exclusion criteria for Peer Mentors Academic Year 2018–2019

All first-year students from the two study campuses who expressed interest to be part of the campus peer mentoring program, who had Android-based smartphones and who gave written consent were recruited to participate. However, students who were repeating their academic year of study and those who had disciplinary cases in college were excluded from the study. This was because the students may have been previously exposed to the campus mentoring and counselling programs and thus introduce a bias to the study.

The study also targeted students who would be role models for positive social behaviour while on campus, therefore those repeating an academic year were deemed to be less favourable role models. These students were trained to be role models and champions for the campaign against alcohol and substance use on campus.

### Peer mentor participant recruitment and training

Advertisements for the recruitment of peer mentors were made through student forums, student internet portals, student leaders, college notice boards, and the distribution of flyers on campus. Interested first-year students in the study campuses were requested to express their interest to be trained as peer mentors by making a written application to the Dean of Students’ office on their campuses. The application criteria were: first-year students who had scored at least a B+ grade at the Kenya certificate of secondary examination and demonstrated involvement in school clubs as this pointed to leadership skills.

The advertisements ran for one month. A total of 120 students responded to this advertisement. Twenty applicants did not qualify for recruitment. 100 applicants were selected by a team led by the principal investigator, leaders of the student mentorship club, and the campus assistant Dean of Students.

**Fig 3:**
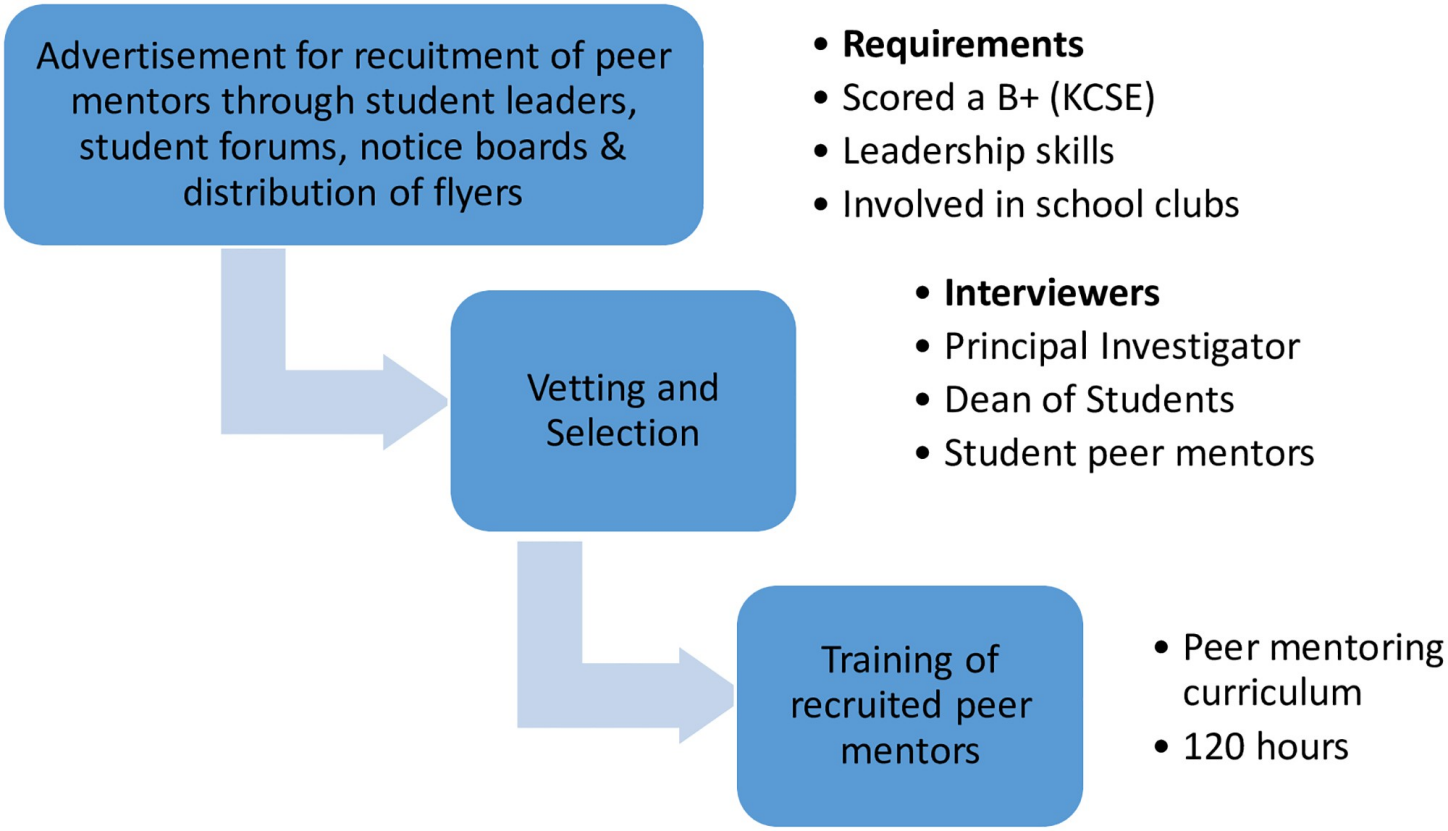
Flowchart of the recruitment procedure.

**Fig 4:**
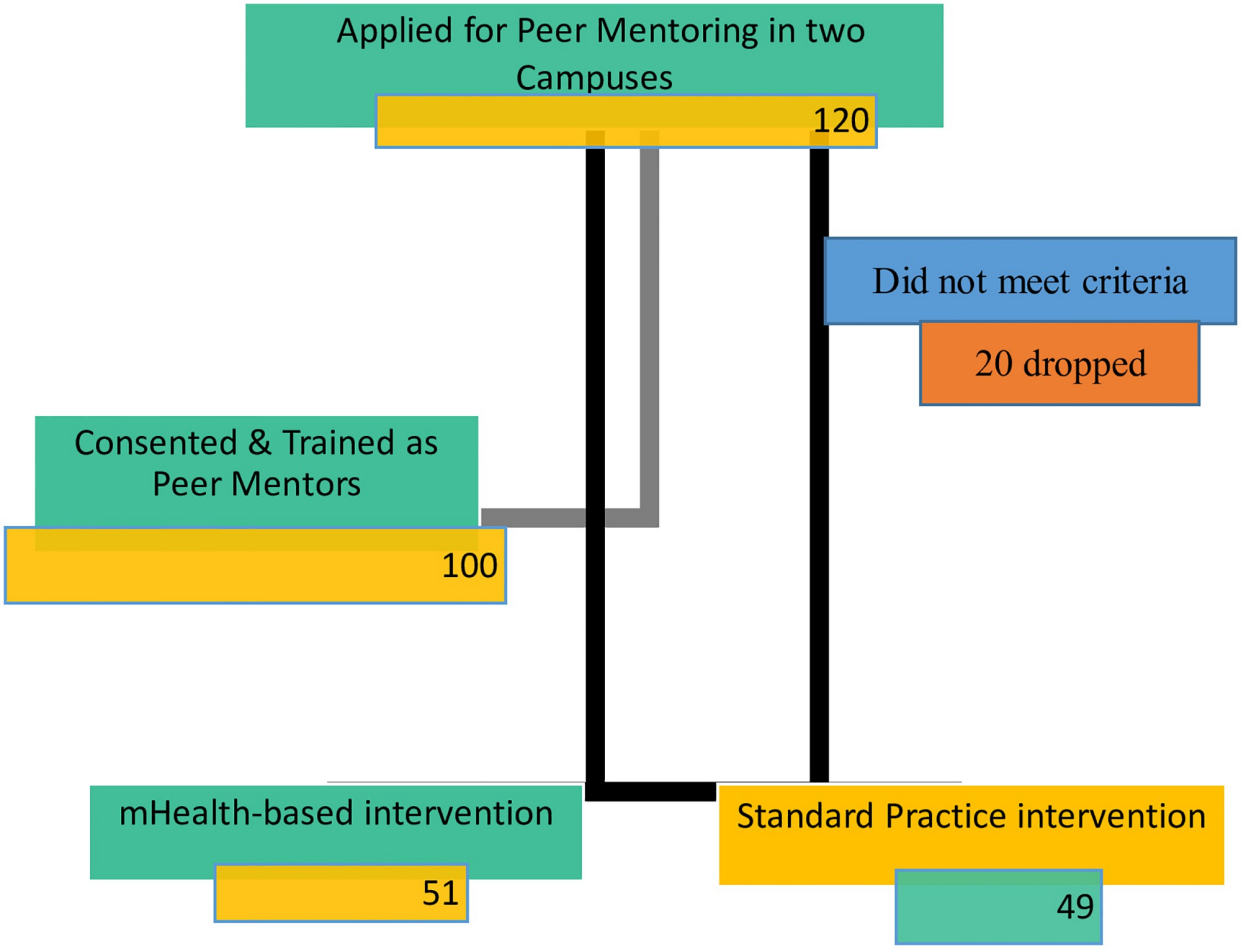
Flowchart of interview and consenting process.

Figs 3 and 4 provide details of the recruitment and training processes. The recruited student peer mentors were then taken through 120 hours of training on various aspects of peer mentoring using a standard curriculum that is in use at the university. This curriculum focuses on alcohol and substance use prevention messages and basic counselling skills. The curriculum has been annexed as S2 File.

After the training, the students were introduced to the details of the study and were taken through the ethical considerations and consenting process. Of the 100 peer mentors who were trained, 51 formed the mHealth-based cohort while 49 formed the standard paper-and-pen cohort. The mHealth cohort also consented to having the mHealth app for peer mentoring installed on their smartphones [29].

The 49 standard paper-and-pen peer mentors were trained to form the comparative control group.

They consented to use the standard practice, as currently is in the University of Nairobi. They used a structured paper-based interview schedule as outlined in S1 File. The interactions of the peer mentors in the standard practice group, with their fellow students, happened face-to-face while they were on campus including in classrooms, halls of residence, and public spaces. Their interactions were recorded in paper diaries and they were required to consult with campus student counsellors as often as necessary.

There was no compensation given to the peer mentors from the standard practice cohort but the peer mentors from the mHealth-based group received internet data bundles of 2 USD every month for the duration of the study.

### Description of the intervention

**Fig 5:**
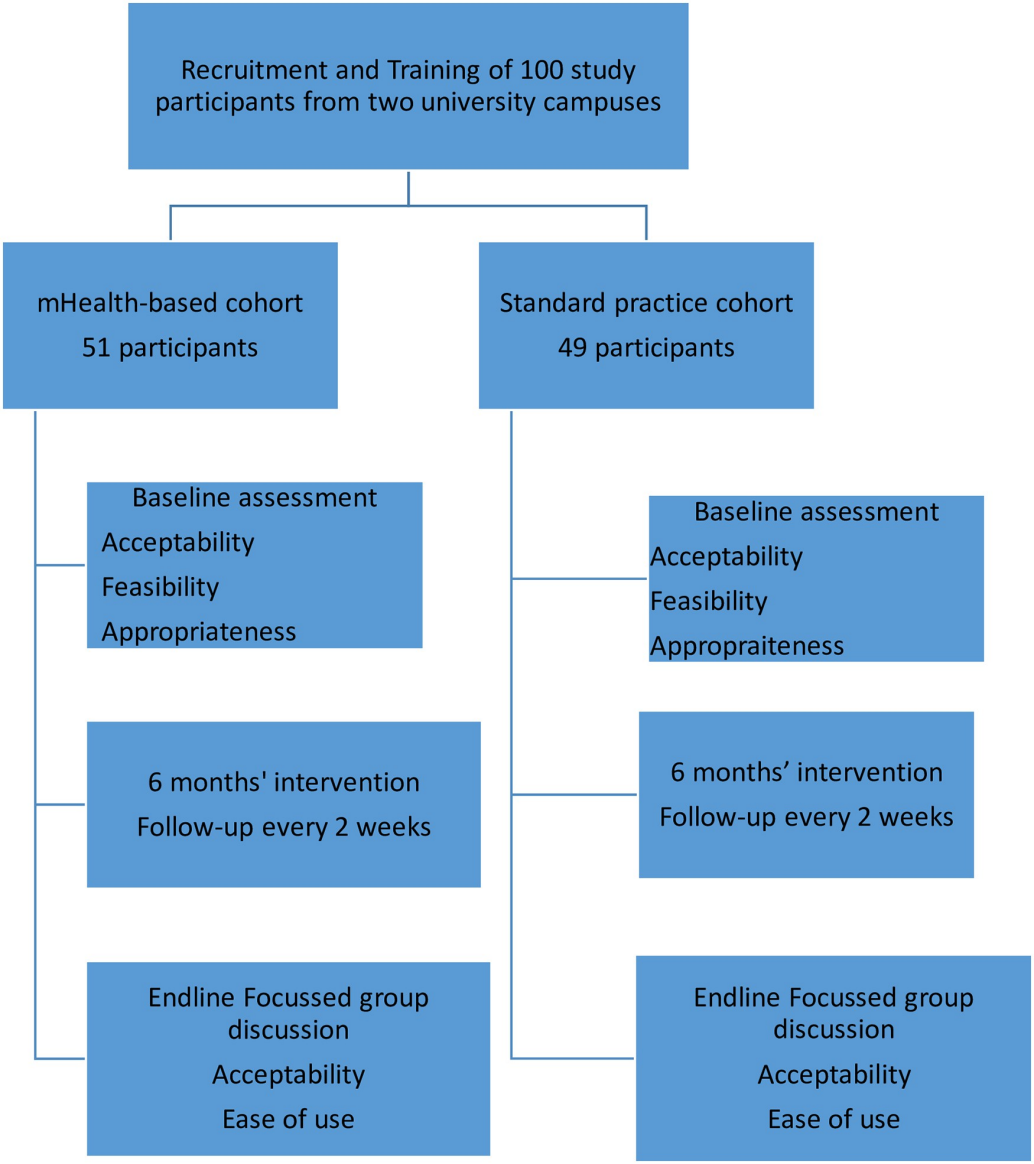
Description of the mHealth-based intervention study.

The Intervention took place between January and July 2019. This 6-month study period was preceded by training of the participants in the use of their respective tools. Peer mentors on one campus were trained to use the mHealth-based intervention tool, which was a digital android-based problem identification, targeted brief intervention, and referral tool. The peer mentors in the comparative group were trained to use the usual standard paper-and-pen-based tool of the university to establish contact with at-risk students and do counselling as trained then send back a paper report to the research team. Both groups had meetings with the research team every two weeks and were followed up for six months. Fig 5 provides more details on this study intervention.

The peer mentors were then trained how to use the application to screen at-risk students and provide brief interventions and referrals as necessary. The mentees would be identified for the mentoring process through various means; 1. The peer mentors were trained in communication and basic counselling skills.

Using these skills, they would then approach fellow students while on campus and explain to them about the peer mentoring program. Peer mentors from both the mHealth-based cohort and the standard practice cohort established contact with their mentees during regular interactions and observation of the behaviour of the mentees while on campus. These behaviours included the frequency of indulging in alcohol and substance use and social interactions of the mentees. The mentors would then ask for the mentee’s consent to participate. 2. Mentees were also referred to the peer mentors by university security personnel, or by faculty members. These were students who had violated university rules, they were given the option to go through the normal university disciplinary processes or to be attached to a peer mentor for guidance. At this point, they had a choice.

Those who opted to be attached to a peer mentor, once contact was established, went through the normal consenting process, they were given the option to opt-out of the program, and only those who gave consent to participate in this program were recruited. There was no coercion on their part. The peer mentor and the mentee mutually agreed on an appropriate time and place for their meeting. 3. The peer mentors held sensitization forums on campus like sports and games, talent shows, and cultural days among other others. These events created opportunities for interaction, and entertainment and to popularize their peer mentoring services, they also wore branded uniforms to advertise their presence on campus. Their fellow students would then voluntarily seek them out when they needed to discuss matters of social concern.

The mHealth-based peer mentoring intervention was advertised through social media platforms like WhatsApp, Facebook, and Instagram which are popular among college students. From these engagements, up to 31% of the mentees in the mHealth-based intervention cohort and 16% in the standard practice cohort made the initiative to seek help from peer mentors.

These interactions happened between the mentor and the mentee face to face at a place and time they agreed on.

Using the mHealth tools (AUDIT and ASSIST) programmed in their smartphones the mentors conducted a structured alcohol and substance use screening process and fill in the responses. After the screening, the mentor gave feedback to the mentee on the scores of their substance use screening.

The mentor interpreted test scores for the mentee and then provided the appropriate intervention. The intervention could be; a brief counselling session, a scheduled follow-up session, and/or referral for further counselling by the campus student counsellor.

This information on the mentor-mentee interaction was relayed to the research team, in real-time through the ODK app.

### Measurements and data collection

The Implementation outcome measures are essential for monitoring and evaluating the success of implementation efforts [31], they are based on the Technology Acceptance Model (TAM) [32]. TAM is a behavioural model which was developed by Davis in 1989 to predict the user acceptance and use of new information technology based on its perceived ease of use and usefulness [33,34]. The TAM tool has been used previously to determine the acceptability of the use of new technology in online teaching systems among students [35,36]. A scoping review of the technology acceptance model found that there are varying definitions of the term technology acceptance. It, therefore, proposed that technology acceptance should be viewed as a staged process, with adapted measurement methods for each stage [37].

TAM has also been studied in resource-limited settings in Uganda and a newer model was proposed for use in such settings [38].

The primary outcomes of the study based on this model included: the implementation measures namely the Acceptability of Intervention (AI), Intervention Appropriateness (IA), Feasibility of Intervention (FI), and the reach of the intervention (e.g., the number of mentees reached and the number of contacts made). These implementation outcomes were assessed using the Acceptability of the Intervention Measure (AIM), the Intervention Appropriateness Measure (IAM), and the Feasibility of the Intervention Measure (FIM). These measures have been reported to have good psychometric properties [30].

Data collection: Peer mentors’ sociodemographic data, including sex, age, the school enrolled, course of study, and mode of sponsorship among other parameters, were collected using a researcher-designed questionnaire.

Initial assessment: 1) AIM 2) IAM 3) FIM. During the interaction between peer mentors and their mentees, the mHealth-based tool collected the following data on the initial contact and follow-up contacts. The number of contacts made, the duration of the interaction, and the presenting problems among other parameters. These data were then transferred directly from the peer mentor’s phone to a central ODK server for storage. Data from the standard practice cohort were collected based on a checklist and data forms developed by the researchers.

The filled checklist and data forms were later collected by the principal investigator who reviewed them for data processing.

### Data management and statistical analysis

Completed data forms were coded and uploaded into the EpiData 3.1 software; they were then checked for completeness and consistency.

Incompletely filled forms were dropped at this point. The cleaned data were then exported to Stata software. All statistical analyses were performed using Stata software version 14.2 Special Edition. Data analysis was done using both descriptive and inferential methods.

The socio-demographic data of peer mentors which contained continuous data were summarized using measures of central statistics and presented as means with standard deviations. While the categorical data were presented as frequencies and proportions.

The Acceptability of Intervention Measure (AIM), Intervention Appropriateness Measure (IAM), and Feasibility of Intervention Measure (FIM) data were analyzed and presented as frequencies and proportions.

### Qualitative data collection

Participants were selected by asking peer mentors in the study who were interested to participate. There were two groups in each arm of the study, each group consisted of 8 to 12 members. The total number of participants was 42.

The first author moderated the group using a focused group discussion (FGD) guide specifically designed to capture their experiences during the peer mentoring process. The FGD is attached (S3 File).

All groups were conducted virtually using zoom and recorded. Data was transcribed from the zoom recording and analysed using NVivo software for themes deductively.

## Results

### 1. Social demographic characteristics of peer mentors

The social demographic distribution of peer mentors is presented in Table 1.

**Table 1:** Social Demographic characteristics of Peer Mentors.

Variables (Social demographics)	mHealth-based Intervention (N = 51)	Standard care cohort (N = 49)	Total (N = 100)	p-value
**Age, yrs.**				0.053
18–21	43 (84.3%)	47 (95.9%)	90 (90.0%)	
22 to 25	8 (15.7%)	2 (4.1%)	10 (10.0%)	
Mean (SD)	19.275 (3.014)	19.510 (1.157)	19.390 (2.291)	0.610
Range	18.000–25.000	18.000–23.000	18.000–25.000	
**Sex**				0.165
Male	31 (60.8%)	23 (46.9%)	54 (54.0%)	
Female	20 (39.2%)	26 (53.1%)	46 (46.0%)	
**Marital status**				0.977
Single	50 (98.0%)	48 (98.0%)	98 (98.0%)	
Not single	1 (2.0%)	1 (2.0%)	2 (2.0%)	
**Residence**				0.020
Campus	39 (76.5%)	47 (95.9%)	86 (86.0%)	
Private	5 (9.8%)	1 (2.0%)	6 (6.0%)	
Home	7 (13.7%)	1 (2.0%)	8 (8.0%)	
**Mode of sponsorship**				0.102
Public-Sponsored	46 (90.2%)	48 (98.0%)	94 (94.0%)	
Private-Sponsored	5 (9.8%)	1 (2.0%)	6 (6.0%)	

One hundred peer mentors participated in the peer mentoring intervention program. Of these, 51 were from the mHealth-based intervention campus. Overall, there were more males, accounting for 54% of the peer mentors. The mean age of the peer mentors was 19.4 (std = 2.3) and it did not differ significantly between the two campuses.

There was a statistically significant difference between the two campuses concerning the place of residence, with more peer mentors in the standard practice cohort who resided on campus (96%) compared to those in the mHealth-based cohort (76%), p = 0.020.

### 2. Social demographic characteristics of the mentees

The results, presented in Table 2, show that the majority of the mentees in both groups were males. In the mHealth-based campus, 87% of the mentees were publicly sponsored compared to 84% on the standard practice campus. Over 70% of the mentees on both campuses resided in campus residences. Most of them were in their second year of study and were single.

**Table 2:** Socio-demographic characteristics of the mentees.

Variable	Campus		p-value
	mHealth-based	Standard practice	
	Freq (Col %)	Freq (Col %)	
**Gender**			0.789
Male	266 (60.5)	59 (59)	
Female	174 (39.5)	41 (41)	
**Year of Study**			<0.001
1	115 (26.1)	9 (9)	
2	164 (37.3)	81 (81)	
3	80 (18.2)	10 (10)	
4	81 (18.4)	0 (0)	
**Mode of sponsorship**			0.421
Public	383 (87)	84 (84)	
Private	57 (13)	16 (16)	
**Marital Status**			0.001
Single	355 (86.2)	98 (98)	
Married/ Cohabiting	57 (13.8)	2 (2)	
**Residence**			0.002
Campus	309 (70.2)	86 (86)	
Private	51 (11.6)	11 (11)	
Home	52 (11.8)	2 (2)	
Other	28 (6.4)	1 (1)	

Comparing the two campuses there was no significant difference in the gender of the students who sought help (p-value = 0.789).

## 3. Peer mentoring intervention acceptability

Both the mHealth-based cohort and the standard practice cohort responded to questionnaires on the acceptability of the respective programs they used for peer mentoring. The acceptability, appropriateness, and feasibility of the peer mentoring programs were assessed using the Acceptability of Intervention questionnaire which is based on the TAM (1989) (S4 File). The results (see Table 3) show that the acceptability of the peer mentoring programs was high in both groups and did not differ significantly between the mHealth-based group (100%) and the standard of care (96%) group. The Appropriateness of the program was rated at 92.2% by peer mentors in the mHealth-based group compared to 98% in the standard practice group. There was no statistically significant difference between the mHealth and standard practice groups in terms of their attitude towards peer mentoring, as 98% of peer mentors from the mHealth group and 100% of those from the standard practice group had a positive attitude towards peer mentoring and intention to use it. Eighty-six (86%) percent of peer mentors from the mHealth-based group found the intervention feasible and reported they had the resources needed to implement the intervention. These resources were: android smartphones, internet connectivity, and technical training.

**Table 3:** Acceptability, APPROPRIATENESS, FEASIBILITY of Intervention Measure (AIM, IAM, FIM tool).

	mHealth Intervention (N = 51)	Standard care (N = 49)	Total (N = 100)	p value
**Acceptability**				0.145
Accept	51 (100.0%)	47 (95.9%)	98 (98.0%)	
Neutral	0 (0.0%)	2 (4.1%)	2 (2.0%)	
**Appropriateness**				0.316
Accept	47 (92.2%)	48 (98.0%)	95 (95.0%)	
Below	2 (3.9%)	0 (0.0%)	2 (2.0%)	
Neutral	2 (3.9%)	1 (2.0%)	3 (3.0%)	
**Feasibility**				0.374
Accept	44 (86.3%)	45 (91.8%)	89 (89.0%)	
Neutral	7 (13.7%)	4 (8.2%)	11 (11.0%)	
**Resources**				0.242
Available	44 (86.3%)	37 (75.5%)	81 (81.0%)	
Not available	4 (7.8%)	4 (8.2%)	8 (8.0%)	
Neutral	3 (5.9%)	8 (16.3%)	11 (11.0%)	
**Usefulness**				0.999
Useful	49 (96.1%)	47 (95.9%)	96 (96.0%)	
Not useful	1 (2.0%)	1 (2.0%)	2 (2.0%)	
Neutral	1 (2.0%)	1 (2.0%)	2 (2.0%)	
**Ease of use**				0.223
Easy	48 (94.1%)	46 (93.9%)	94 (94.0%)	
Not easy	3 (5.9%)	1 (2.0%)	4 (4.0%)	
Neutral	0 (0.0%)	2 (4.1%)	2 (2.0%)	
**Attitude towards peer mentoring**				0.325
Positive	50 (98.0%)	49 (100.0%)	99 (99.0%)	
Negative	1 (2.0%)	0 (0.0%)	1 (1.0%)	

*Significance at p≤0.05

### Outcomes of the qualitative interviews

Themes that emerged included; Opportunities for personal learning, growth, and development for the peer mentors, enhanced communication skills for peer mentors, learning of life skills, and improved self-awareness for peer mentors.

The results showed that there was high acceptability of the peer mentoring program by the student community. The peer mentors who participated in this study reported that they got personal benefits from their participation. Most of them reported that they had gained new information and improved their communication and interpersonal skills. One peer mentor had this to say: “Through this program, I improved on my communication skills, I gained skills and confidence on how to start a conversation.” (Female peer mentor 22 years).

Another one said: “I enjoyed helping my peers, I mastered all the mentoring process and the questions and the materials I had on Alcohol and Drug Abuse education came in very handy.” (male peer mentor, 22 years). Moreover, peer mentors reported that they learned life skills including time management, self-control as well as self-organization. Overall peer mentors reported that their campus life had improved due to their involvement in the peer mentoring activities. They were satisfied with their performance and they expressed willingness to continue with the peer mentoring activities on campus. One student reported that: “Students still seek me out for mentoring, it should continue and more to more campuses” (Male peer mentor 20 years) while another one reported that: “I desire to continue with this wonderful work, not to leave it” (Female peer mentor 19 years). While yet another was categorical that the program should continue: “This program should continue; it should not end here.” (Male peer mentor 24 years).

### 4: Intervention reach and the problems as identified by the peer mentors

A summary of the reach attained and problems identified by the peer mentors are presented in Table 4. A total of 540 (60% males) of the mentees were reached on the two campuses.

**Table 4:** Contacts made and the problems as identified by the peer mentors.

Variable	Campus		p-value
	mHealth-based Intervention	Standard care intervention	
	Freq (Col %)	Freq (Col %)	
**How contact was made**			<0.001
Mentor initiated	186 (42.5)	70 (70)	
Mentee initiated	135 (30.8)	16 (16)	
External referral	117 (26.7)	14 (14)	
**Problems identified**			
**Alcohol use**			<0.001
No	224 (50.9)	74 (74)	
Yes	216 (49.1)	26 (26)	
**Other drugs use**			<0.001
No	315 (71.6)	90 (90)	
Yes	125 (28.4)	10 (10)	
**Finance problems**			0.004
No	349 (79.3)	66 (66)	
Yes	91 (20.7)	34 (34)	
**Intimate Relationships**			0.376
No	356 (80.9)	77 (77)	
Yes	84 (19.1)	23 (23)	
**Family relationships**			0.837
No	402 (91.4)	92 (92)	
Yes	38 (8.6)	8 (8)	
**Academic difficulties**			0.112
No	387 (88)	82 (82)	
Yes	53 (12)	18 (18)	
**Sexual Assault**			0.095
No	428 (97.3)	100 (100)	
Yes	12 (2.7)	0 (0)	
**Stress**			0.321
No	366 (83.2)	79 (79)	
Yes	74 (16.8)	21 (21)	

*Significance at p≤0.05

In the mHealth-based cohort, the mentors reached a total of 440 mentees, while in the standard practice cohort 100 mentees were reached. The mean number of mentees per mentor in the mHealth cohort was 8.62 (std = 13.04) with a range of (1,76) while that in the standard practice cohort was 8.33 (std = 4.40) with a range of (3,19). While the mean number of mentees reached did not differ significantly when we compared the two groups (P-value = 0.874), the results show that the peer mentor who reached most mentees in the mHealth-based cohort reached almost 4 times more mentees than the counterpart in the standard practice cohort.

As pertains to the problems that were presented to the peer mentors, at the end of the interventions, up to 49% of the mentees in the mHealth-based intervention cohort presented with alcohol-related problems while 28% had problems with a combination of alcohol and other drugs.

In the standard practice cohort, 26% of the mentees had alcohol-related problems while 10% had a combination of alcohol and other drug problems. The results (see Table 4 above) show that there was a significant difference between the two campuses (p-value = 0.001).

Mentees from the mHealth-based campus had a higher pattern of harmful use of alcohol at 19% as compared to 5% of mentees from the standard care campus as exemplified by scores of 8–14 points on the AUDIT screening. There was a higher proportion of mentees with alcohol-related problems on the mHealth-based campus 93% (n = 237) as compared to 20% (n = 100) on the standard practice campus. Similarly, 40% of mentees from the mHealth-based campus needed intervention for substance use disorders compared to 5% of those from the standard practice campus (Table 5).

**Table 5:** Risk assessment for Alcohol Use Disorders by mentees (AUDIT).

Audit	Campus		P-value
	mHealth-based intervention	Standard care intervention	
	Freq (Col %)	Freq (Col %)	
**Harmful**			<0.001
No	264 (81.5)	95 (95.0)	
Yes	60 (18.5)	5 (5.0)	
**Dependence**			<0.001
No	138 (58.2)	95 (95.0)	
Yes	99 (41.8)	5 (5.0)	
**Alcohol-related problems**			<0.001
No	16 (6.7)	80 (80.0)	
Yes	221 (93.3)	20 (20.0)	
**Risk**			<0.001
Low	34 (14.3)	83 (83.0)	
Risky	76 (32.1)	8 (8.0)	
High	32 (13.5)	4 (4.0)	
Need Intervention	95 (40.1)	5 (5.0)	

*Significance at p≤0.05

## Discussion

The results of this study show a high level of acceptance of the use of mHealth-based intervention strategies by university students who served as peer mentors across the cohorts. These results are similar to a study by Marsch and Borodovsky [27], which showed that the use of technology provides an acceptable avenue to deliver evidence-based alcohol and drug interventions to young adults as compared to face-to-face programs. The evidence generated from this study provides arguments in favour of mHealth-based interventions and the model can be useful in designing similar interventions. The mHealth-based approach has the potential to overcome the challenges associated with the current face-to-face models of delivering alcohol and substance abuse intervention strategies like confidentiality and stigma [39].

The peer mentors who used the mHealth-based tool mentored 4 students for every one student mentored by those using the standard practice. This difference may have been because peer mentors who used the mHealth-based tool were more motivated about the use of a technology-based tool and this may have made them more confident in their intervention. They also received 2 USD monthly bundles and this may have been a motivator too. These results could also be because the mHealth app platform felt somehow more confidential, so mentees in the mHealth-based intervention group who misused alcohol and other substances felt more comfortable reporting (or even enrolling) in a digital-based intervention than in the paper-based intervention.

It could however also be because the mentors who used the standard practice failed to document and report all their interventions which would not be surprising when you consider the tedium of paper reports. Overall, these study results indicate that, although the practice of peer mentoring is acceptable to college students, its actual performance is dependent on the mode of delivery.

Moreover, these findings affirm the evidence that current substance use intervention programs delivered via face-to-face interactions between the youth and a counsellor reach fewer students; with only 10–15% of adolescents receiving the interventions they need for their substance use disorders [40]. The mHealth-based peer mentoring intervention was advertised through social media platforms like WhatsApp, Facebook, and Instagram which are popular among college students. This accounted for its success in reaching more students. The results of this study are similar to the findings of the systematic review of what works in mHealth-based interventions which found that there are many positive outcomes associated with mHealth as an innovative technology to deliver health services [41]. These study findings represent a significant positive step in the prevention of substance use among college students.

The results of the study provide evidence that the use of the mHealth-based tool for peer mentoring improves early identification and interventions for harmful alcohol and substance use among college students.

Studies on substance use have shown that college students initiate alcohol and marijuana use most frequently while in college [42].

Furthermore, alcohol and substance use has been documented to start and escalate when students transition from one stage of their education to another, especially during matriculation and school holidays [43]. The peer mentors using mHealth-based intervention were more likely to identify alcohol use problems and other psychoactive substance use problems at (49% and 28%) respectively, as compared to the standard practice (26%, 10%), while the standard practice group was more likely to identify the other non-substance use related problems like problems related to finances (34%). This may be an indication that the mHealth-based intervention was more sensitive and better than the standard paper-based tool at identifying these more specialized problems.

It is to be noted that non-specialised persons often feel incompetent to deal with any type of problem was probably in this case an indicator that using a mHealth app can overcome this deterrent even in other settings. There may also be differences in the type of students attending these two campuses concerning their areas of study. The mHealth-based campus has science-based courses while the standard practice campus teaches pedagogy-based courses. The students in these two campuses may have different intensities in their coursework. This may show in the amount of time they have to engage in leisure activities, including the consumption of alcohol and other substances.

This study also provided evidence that peer mentors can be used to communicate behaviour change messages among their peers about alcohol and drug use prevention.

These study findings are similar to those by Delacruz et al. [38], who reported that peer mentors play a significant role in shaping new sets of skills, knowledge, and understanding. Peer mentoring will help to surmount the barrier of the stigma associated with seeking help for the use of alcohol and psychoactive substances from more formal and traditional counselling services [44].

Peers interact in informal sessions while on campus, in halls of residence, or during recreational times thus giving multiple opportunities for mentors to initiate interactions with mentees in a non-threatening manner.

### Strengths of the study

This study adds to the evidence for the implementation of mHealth-based peer mentoring intervention for alcohol and substance abuse prevention among university students in a developing country. Furthermore, the results of this study have positive implications for strengthening interventions on substance use prevention through the use of a popular platform with a wider reach, among university students and will serve as a reference point for future comparative studies and scale-up interventions.

### Limitations of the study

This study was done on two campuses of a single public university in Kenya, the study results, therefore, cannot necessarily be generalizable to the national or global university community. Furthermore, the campuses under study did not teach similar courses which means that the academic challenges experienced by students on these campuses and how they cope with them, concerning harmful use of alcohol and drugs and therefore the feasibility-acceptability of alcohol and drug abuse prevention interventions, are not similar enough to allow for a direct correlation. It is important in future studies to explore the impact of these apparent differences on the behaviour of students regarding psychoactive substance use.

### Conclusions and recommendations

The mHealth-based tool had a high level of utility among student peer mentors. The peer mentors’ approval rating of the use mHealth for alcohol and substance abuse prevention was high. mHealth-based interventions provide an opportunity to rapidly expand access and availability of evidence-based alcohol and substance abuse interventions for youth. The study results demonstrated an advantage of the mHealth-delivered intervention over the standard paper-based intervention. The mHealth-based cohort reached a larger number of college students. At the same time, mHealth-based interventions can be used in combination with face-to-face interventions or as stand-alone interventions, therefore, being more versatile compared to paper-based interventions.

It is therefore important to use mHealth-based interventions more to provide interventions to young people as they have the greatest uptake and interest in using technology.

While the focus of the present study was on implementation issues, further studies are recommended to evaluate the effectiveness of the mHealth-based intervention in initiating positive behaviour change towards alcohol and substance abuse prevention among university students. It is important to study how the socio-demographic characteristics of the peer mentors influence their perceptions of the mHealth-based interventions. Also, future research could seek to understand the reasons for the differences between the uptake of mHealth delivered peer mentoring as compared to the standard practice.

As well it would be important in future research to test the mHealth-based versus standard practice interventions using participants of the same population (e.g., campus) to see if mentees are still more likely to self-report higher substance use on the mHealth app than in-person.

## Supporting information

S1 File Paper-based tool used by the standard practice cohort. (ZIP)

S2 File Peer mentoring curriculum. (ZIP)

S3 File Focused group discussion guide. (ZIP)

S4 File Acceptability of intervention measure questionnaire. (ZIP)

S1 Data Standard practice peer mentors. (XLSX)

S2 Data Peer mentors mHealth cohort. (XLSX)
