# Supplementary figures and images for: Student peer mentoring: Feasibility and acceptability of mHealth-based tool for alcohol and substance abuse prevention by peer mentors at a university in Kenya
Source: PLOS Digit Health. 2023 Jan 12;2(1):e0000177. doi: 10.1371/journal.pdig.0000177 (PMC9931292; doi:10.1371/journal.pdig.0000177)

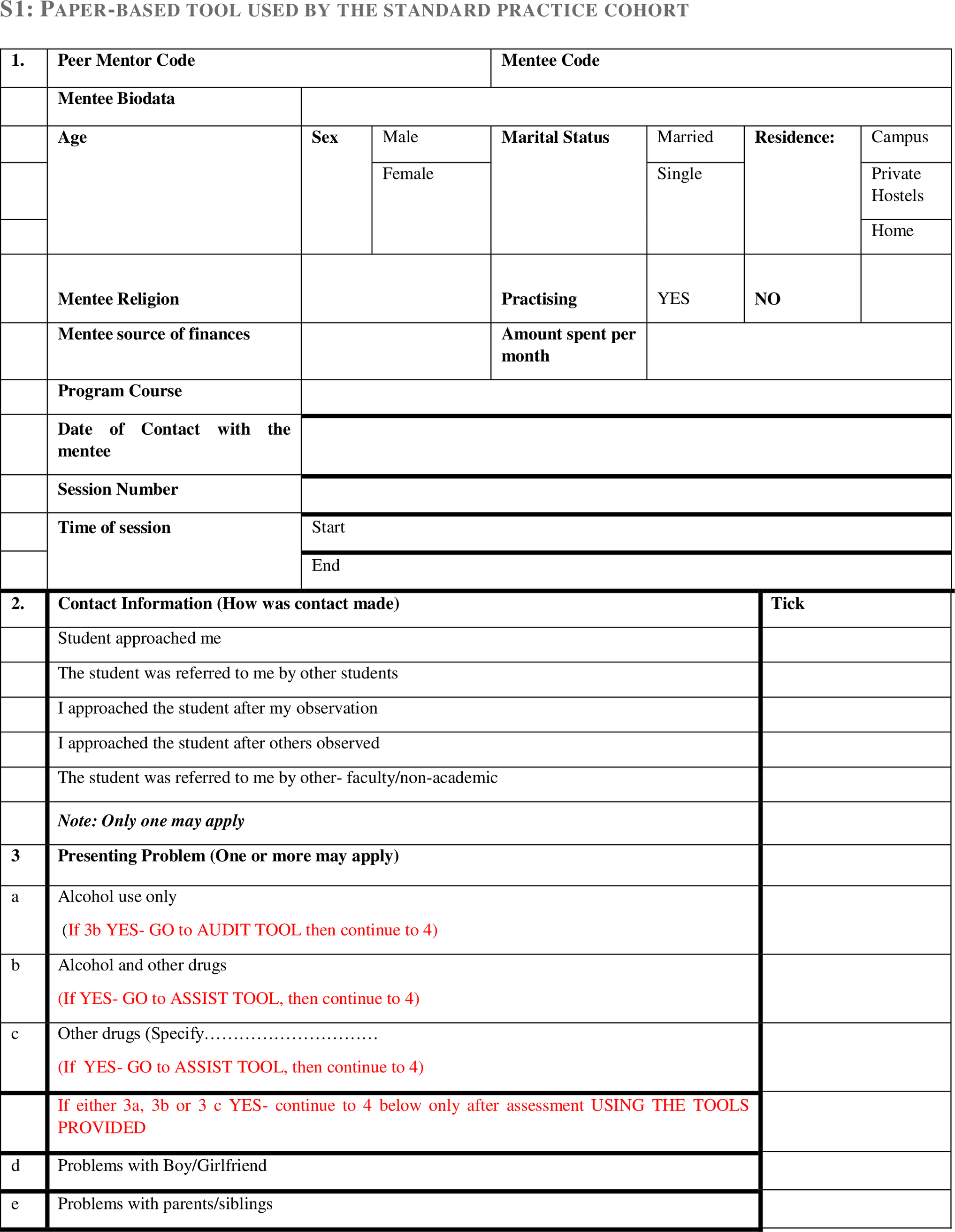

Supplement: S1 File — (ZIP) [file pdig.0000177.s001.zip › PACE Corrected/S1_STANDARD COHORT INTERVIEW GUIDE.tif]

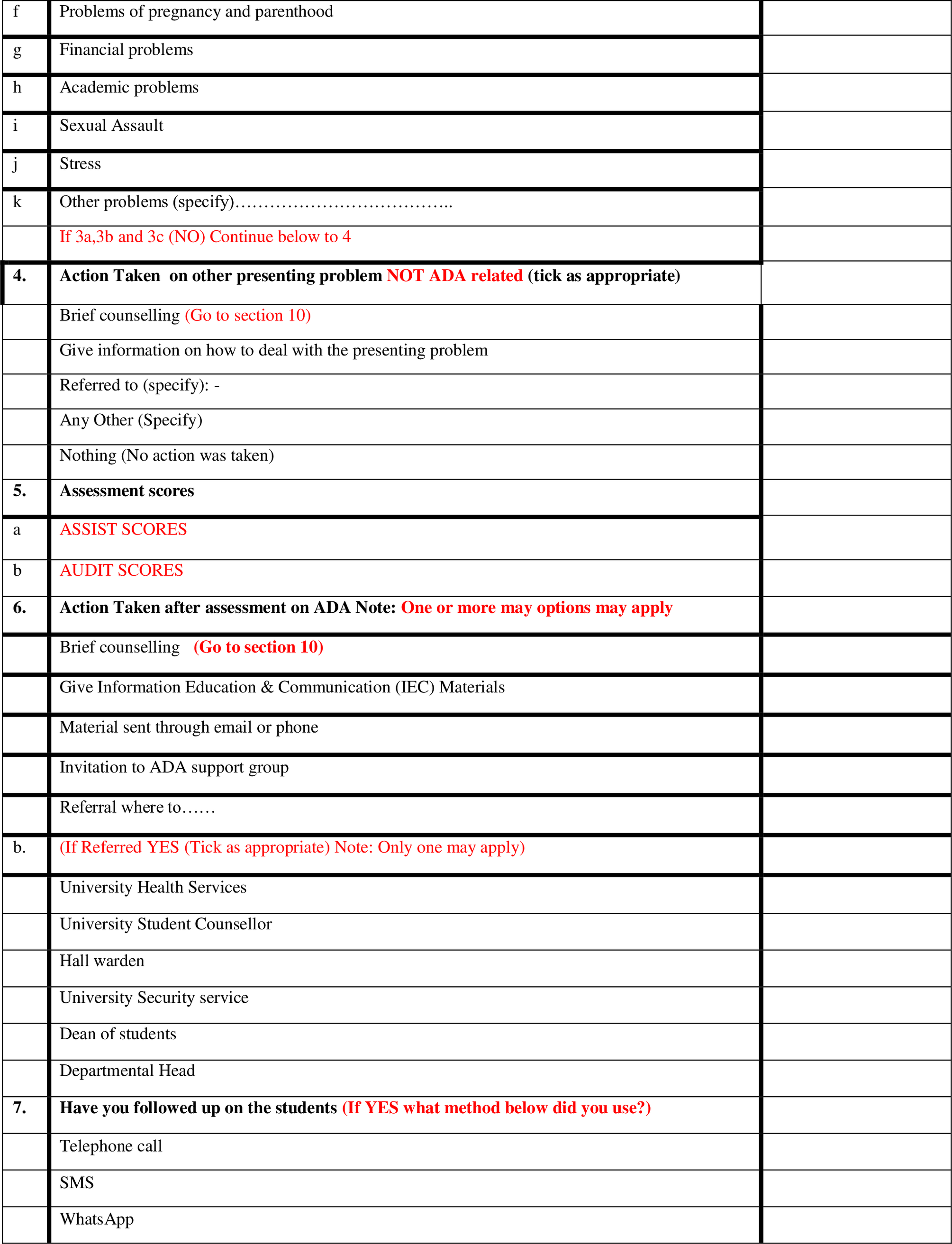

Supplement: S1 File — (ZIP) [file pdig.0000177.s001.zip › PACE Corrected/S1_STANDARD COHORT INTERVIEW GUIDE.tif]

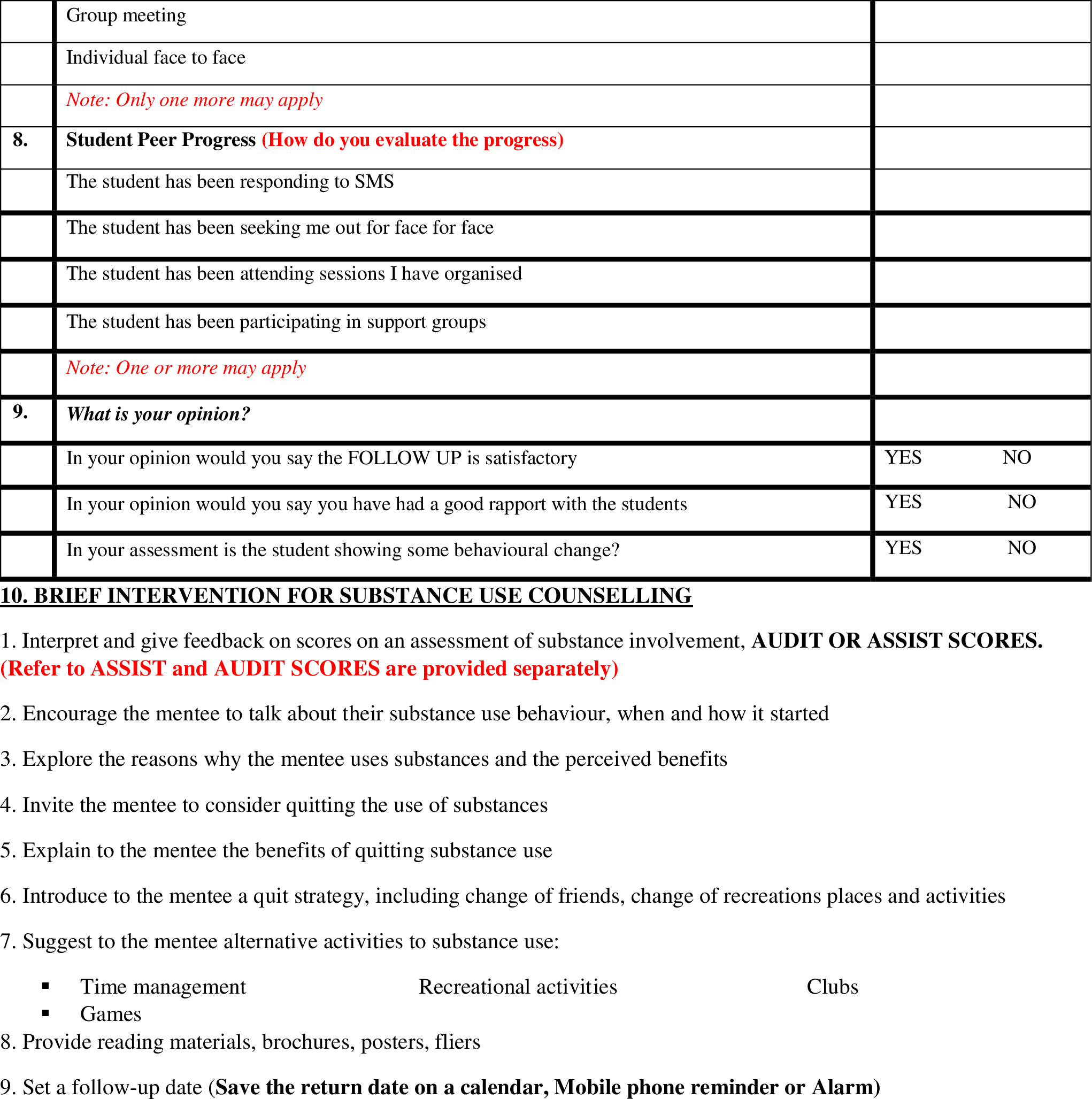

Supplement: S1 File — (ZIP) [file pdig.0000177.s001.zip › PACE Corrected/S1_STANDARD COHORT INTERVIEW GUIDE.tif]

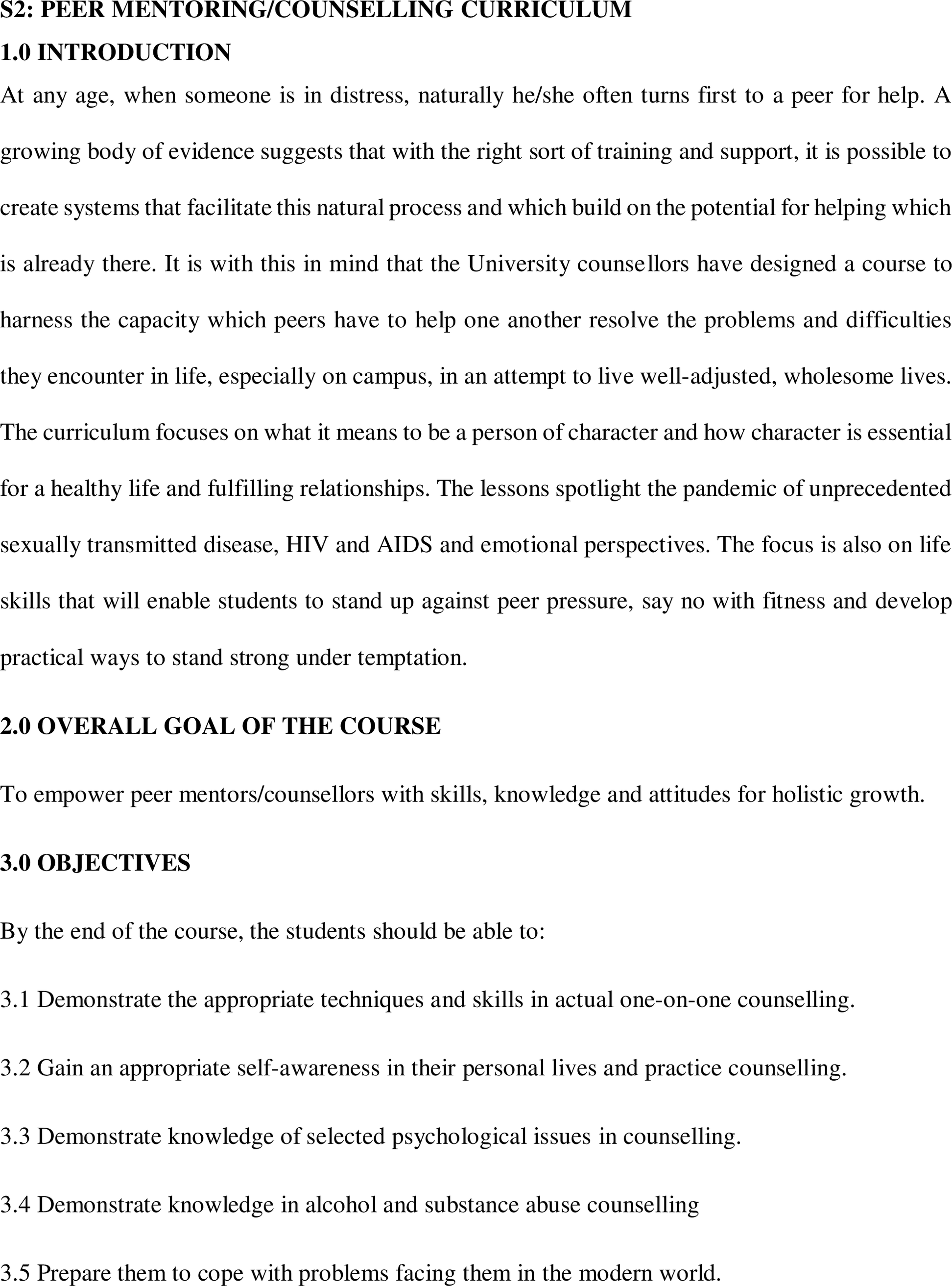

Supplement: S2 File — (ZIP) [file pdig.0000177.s002.zip › PACE Corrected/S2_PEER MENTORING-CURRICULUM.tif]

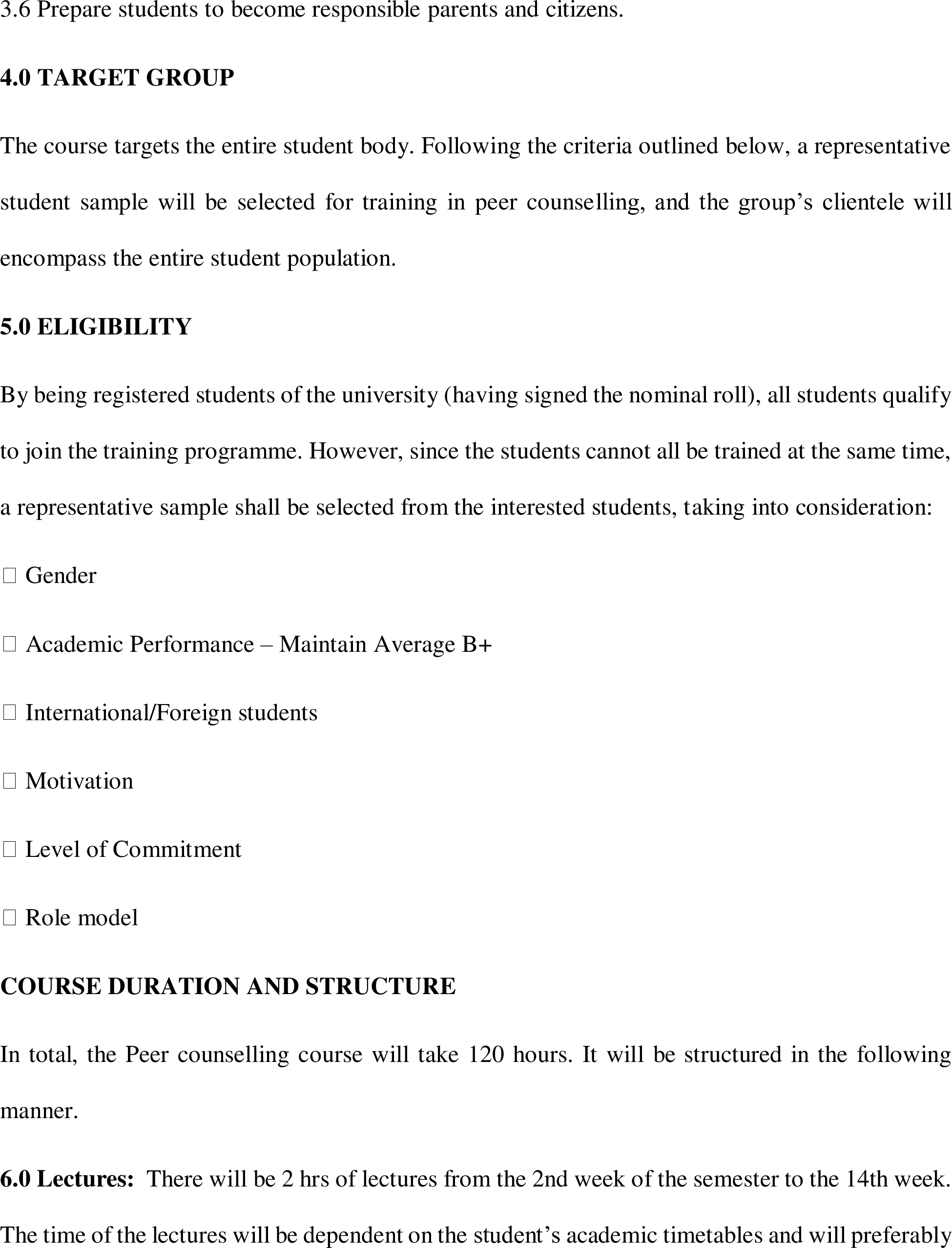

Supplement: S2 File — (ZIP) [file pdig.0000177.s002.zip › PACE Corrected/S2_PEER MENTORING-CURRICULUM.tif]

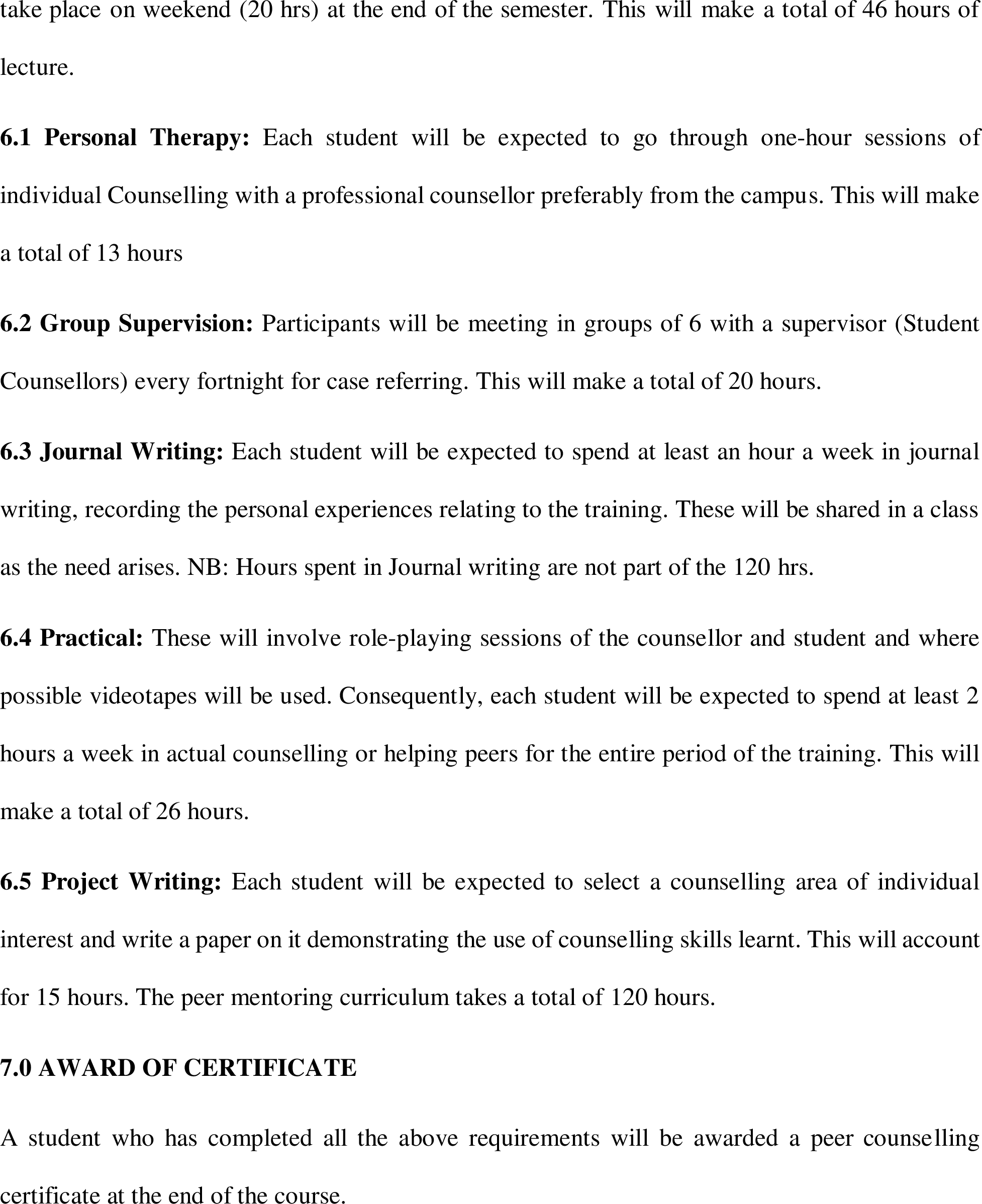

Supplement: S2 File — (ZIP) [file pdig.0000177.s002.zip › PACE Corrected/S2_PEER MENTORING-CURRICULUM.tif]

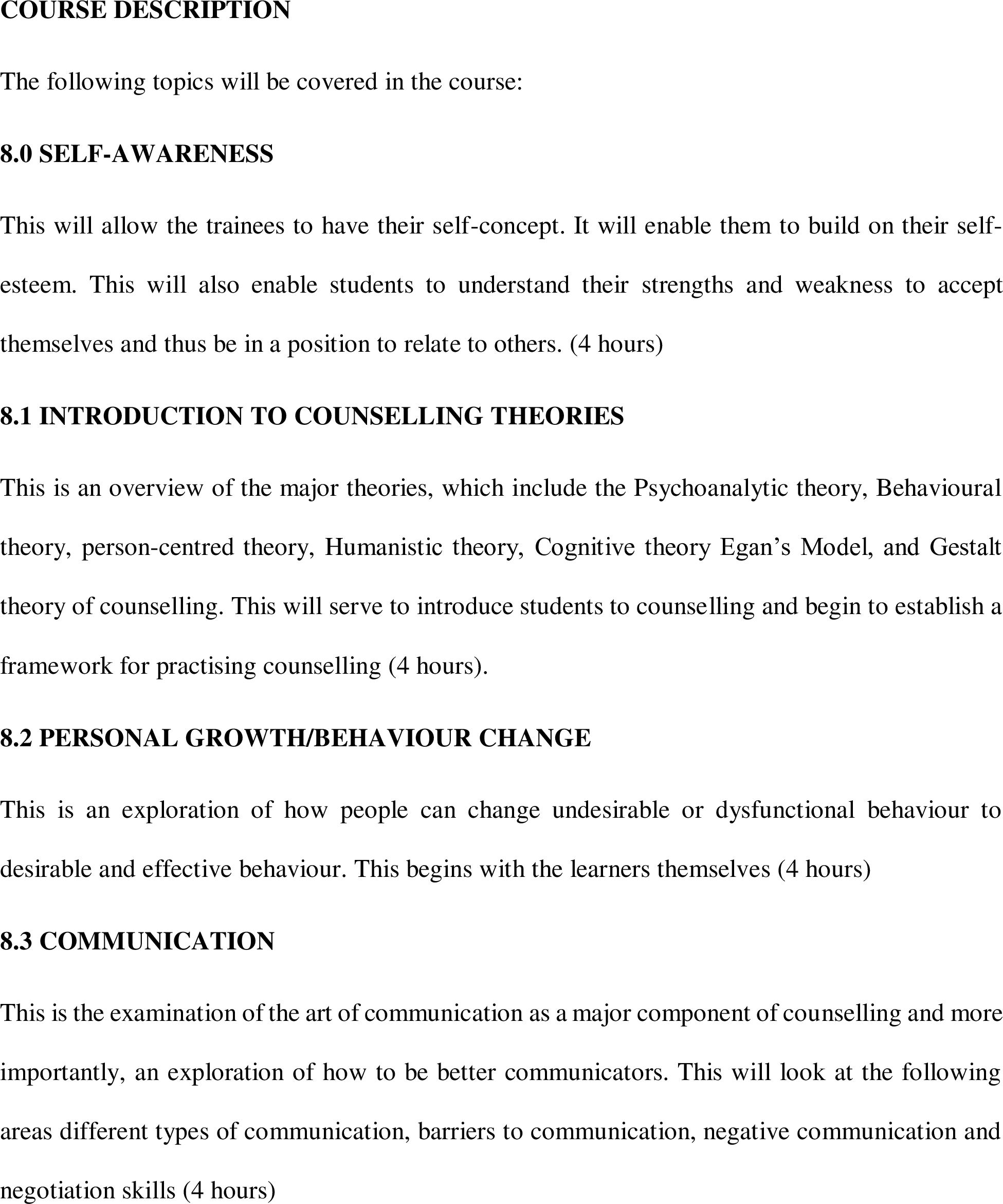

Supplement: S2 File — (ZIP) [file pdig.0000177.s002.zip › PACE Corrected/S2_PEER MENTORING-CURRICULUM.tif]

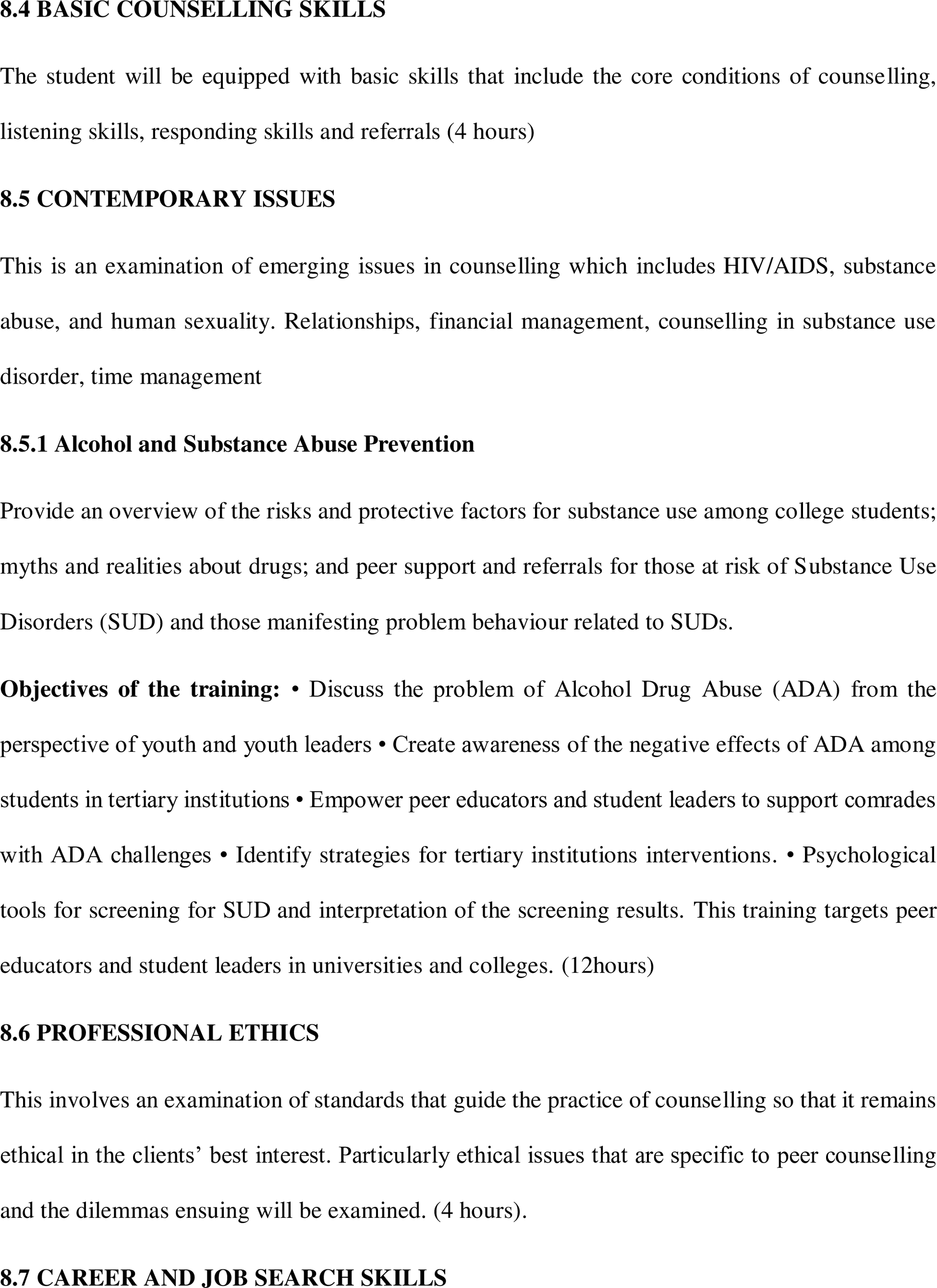

Supplement: S2 File — (ZIP) [file pdig.0000177.s002.zip › PACE Corrected/S2_PEER MENTORING-CURRICULUM.tif]

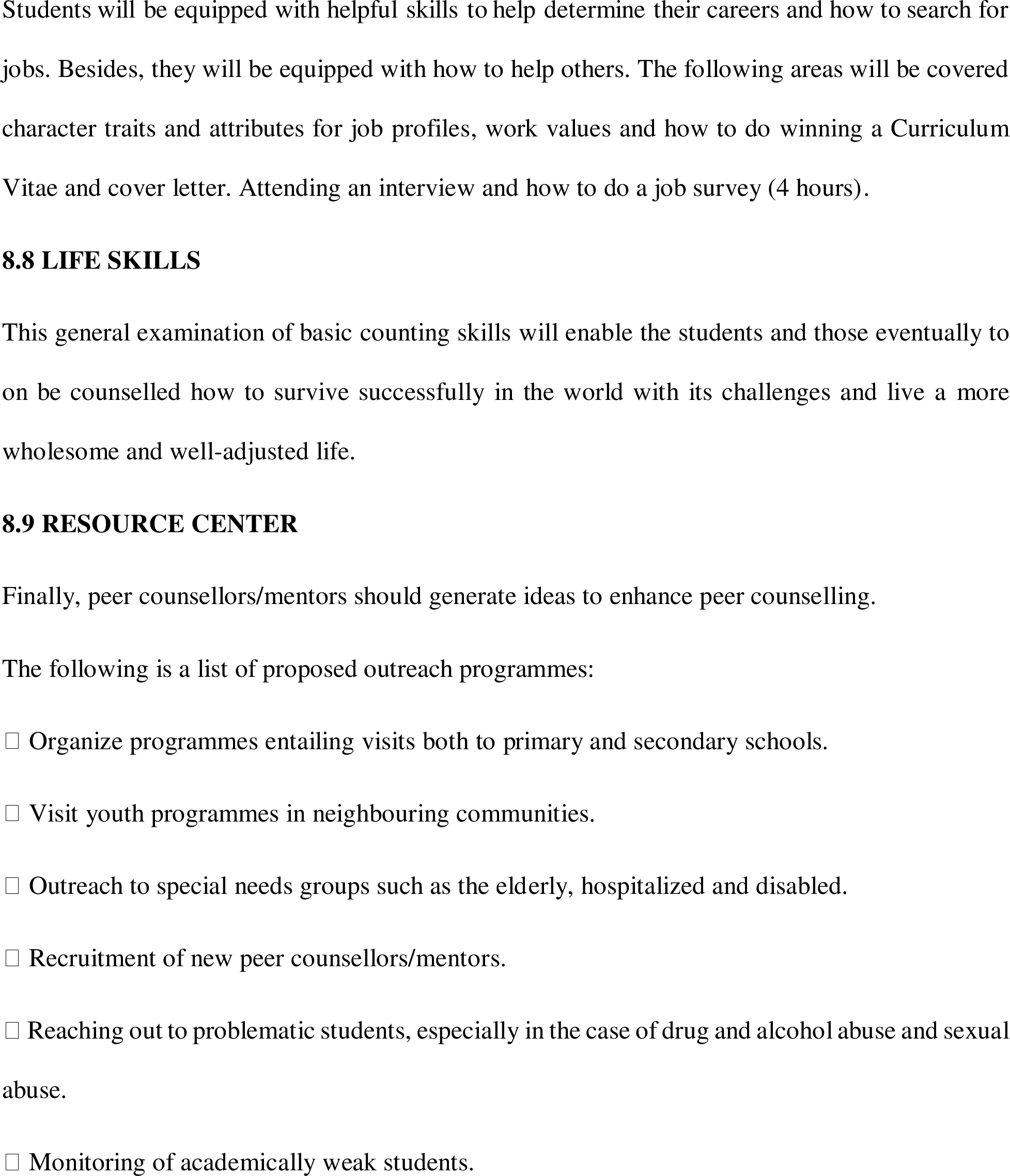

Supplement: S2 File — (ZIP) [file pdig.0000177.s002.zip › PACE Corrected/S2_PEER MENTORING-CURRICULUM.tif]

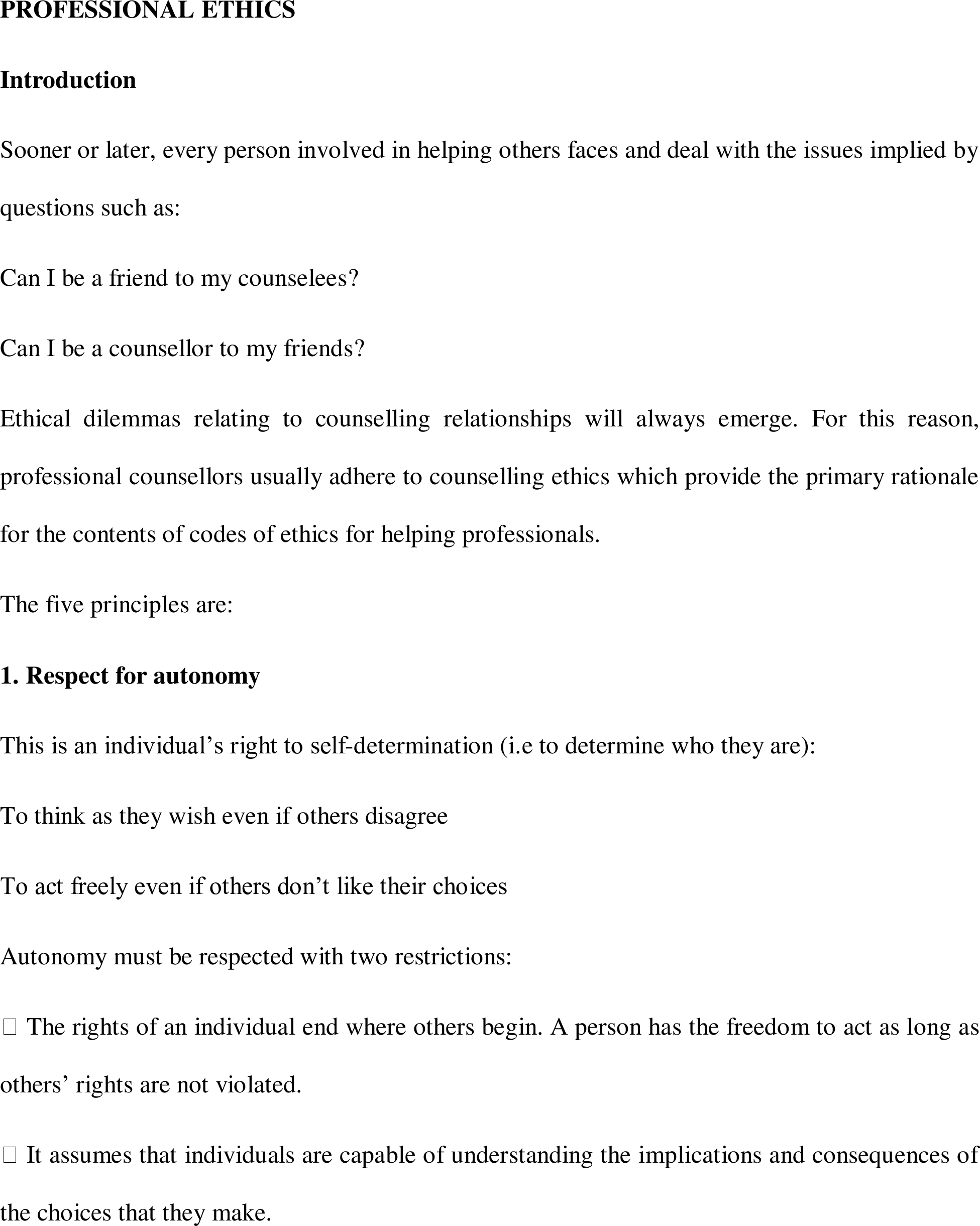

Supplement: S2 File — (ZIP) [file pdig.0000177.s002.zip › PACE Corrected/S2_PEER MENTORING-CURRICULUM.tif]

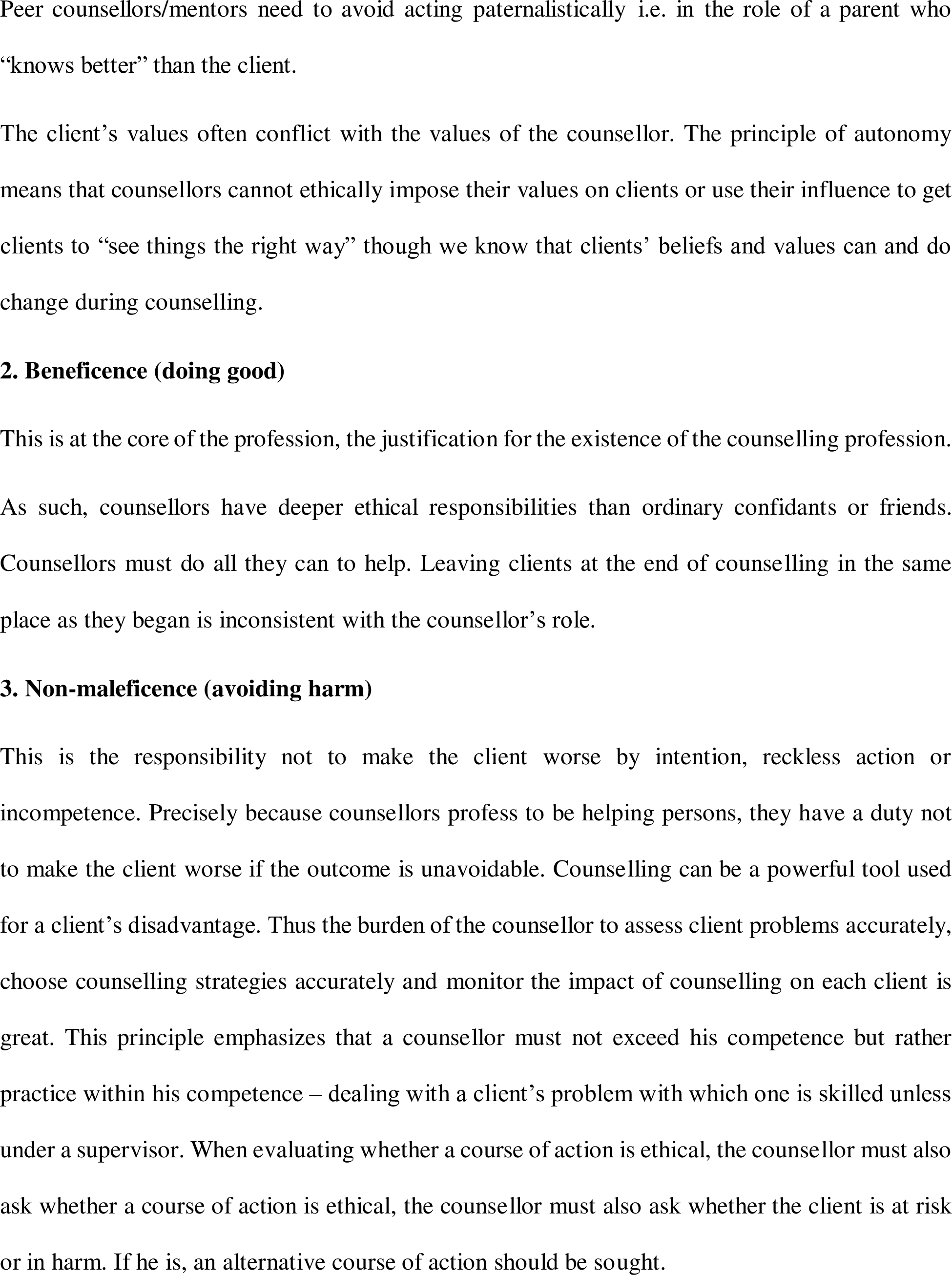

Supplement: S2 File — (ZIP) [file pdig.0000177.s002.zip › PACE Corrected/S2_PEER MENTORING-CURRICULUM.tif]

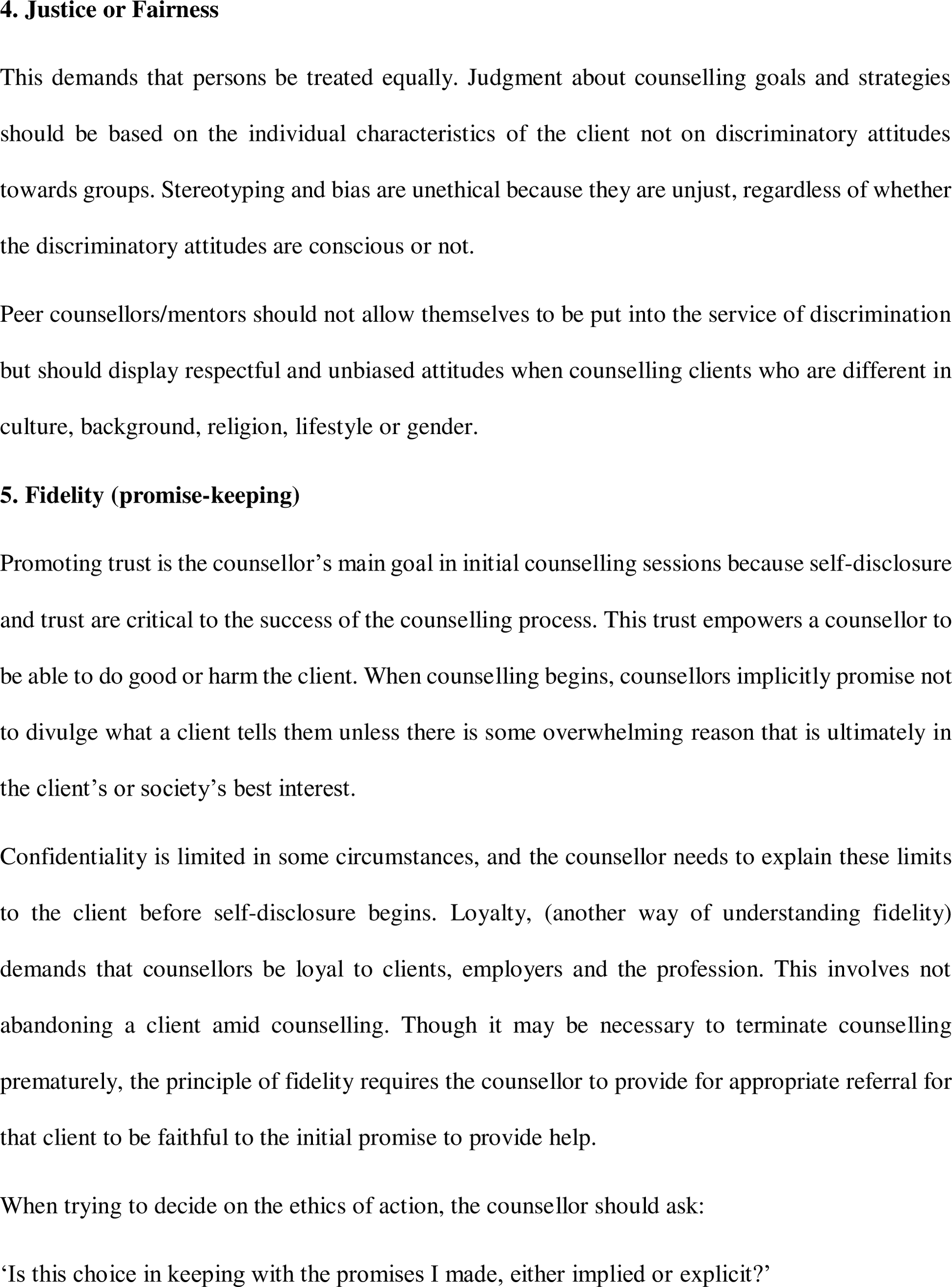

Supplement: S2 File — (ZIP) [file pdig.0000177.s002.zip › PACE Corrected/S2_PEER MENTORING-CURRICULUM.tif]

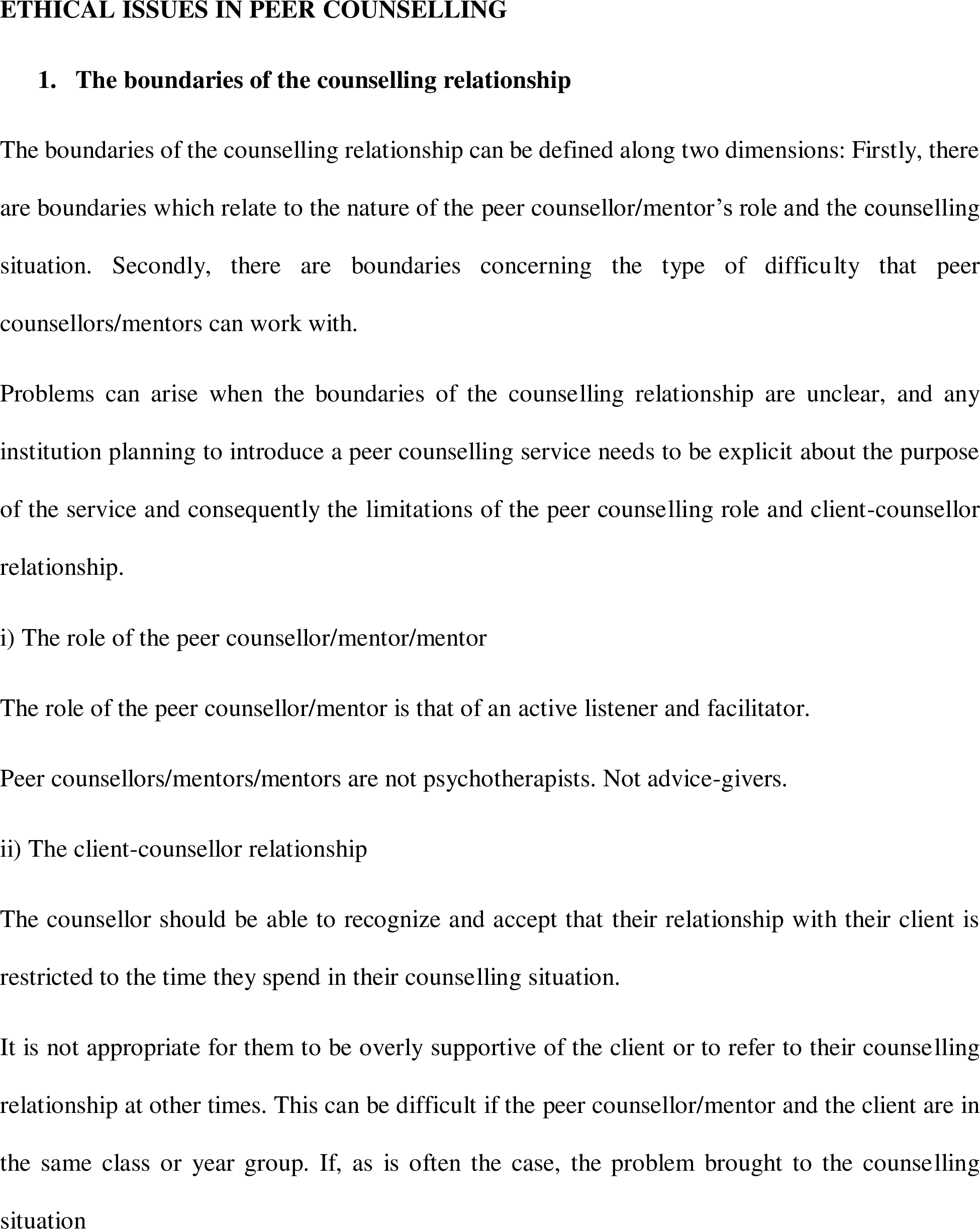

Supplement: S2 File — (ZIP) [file pdig.0000177.s002.zip › PACE Corrected/S2_PEER MENTORING-CURRICULUM.tif]

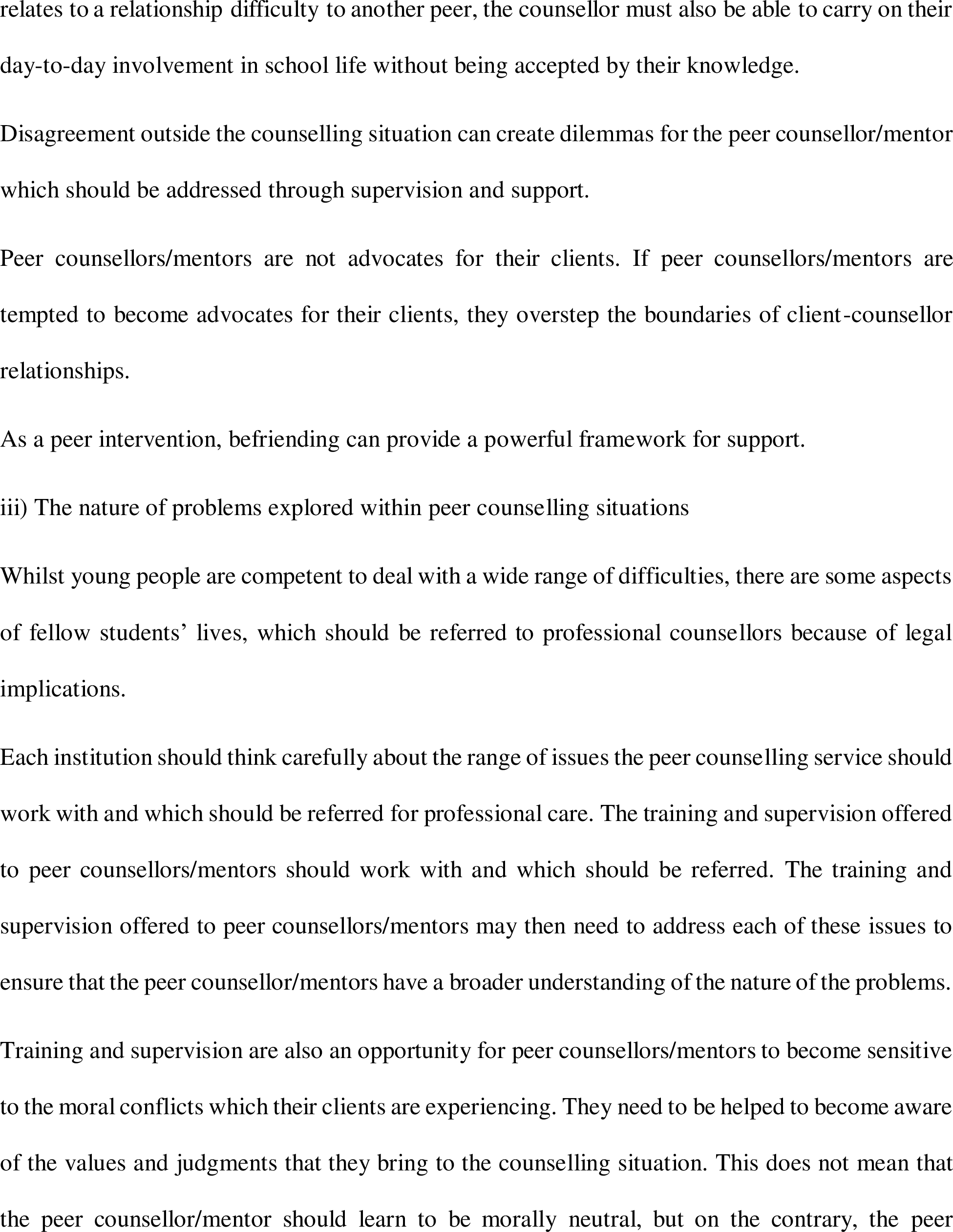

Supplement: S2 File — (ZIP) [file pdig.0000177.s002.zip › PACE Corrected/S2_PEER MENTORING-CURRICULUM.tif]

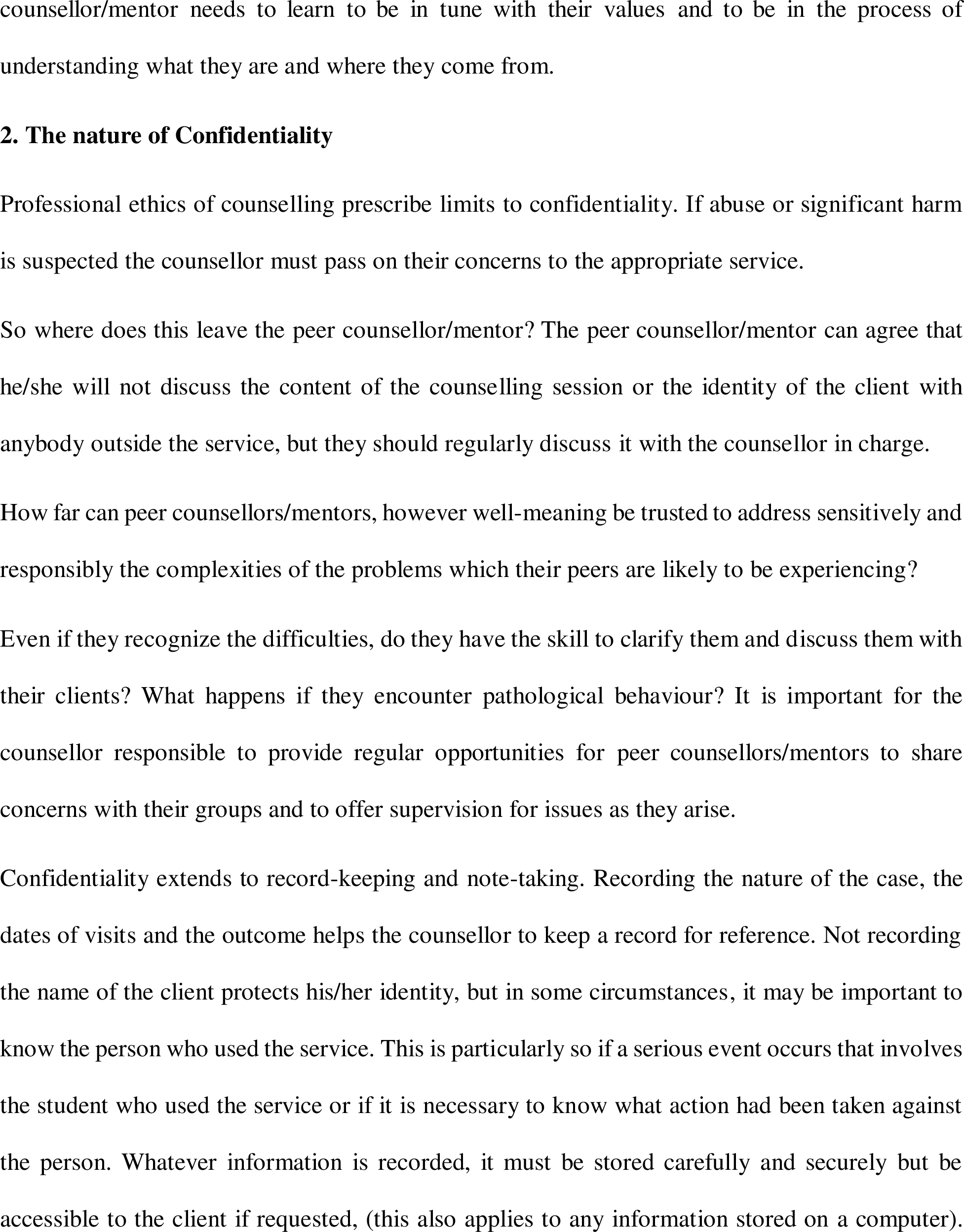

Supplement: S2 File — (ZIP) [file pdig.0000177.s002.zip › PACE Corrected/S2_PEER MENTORING-CURRICULUM.tif]

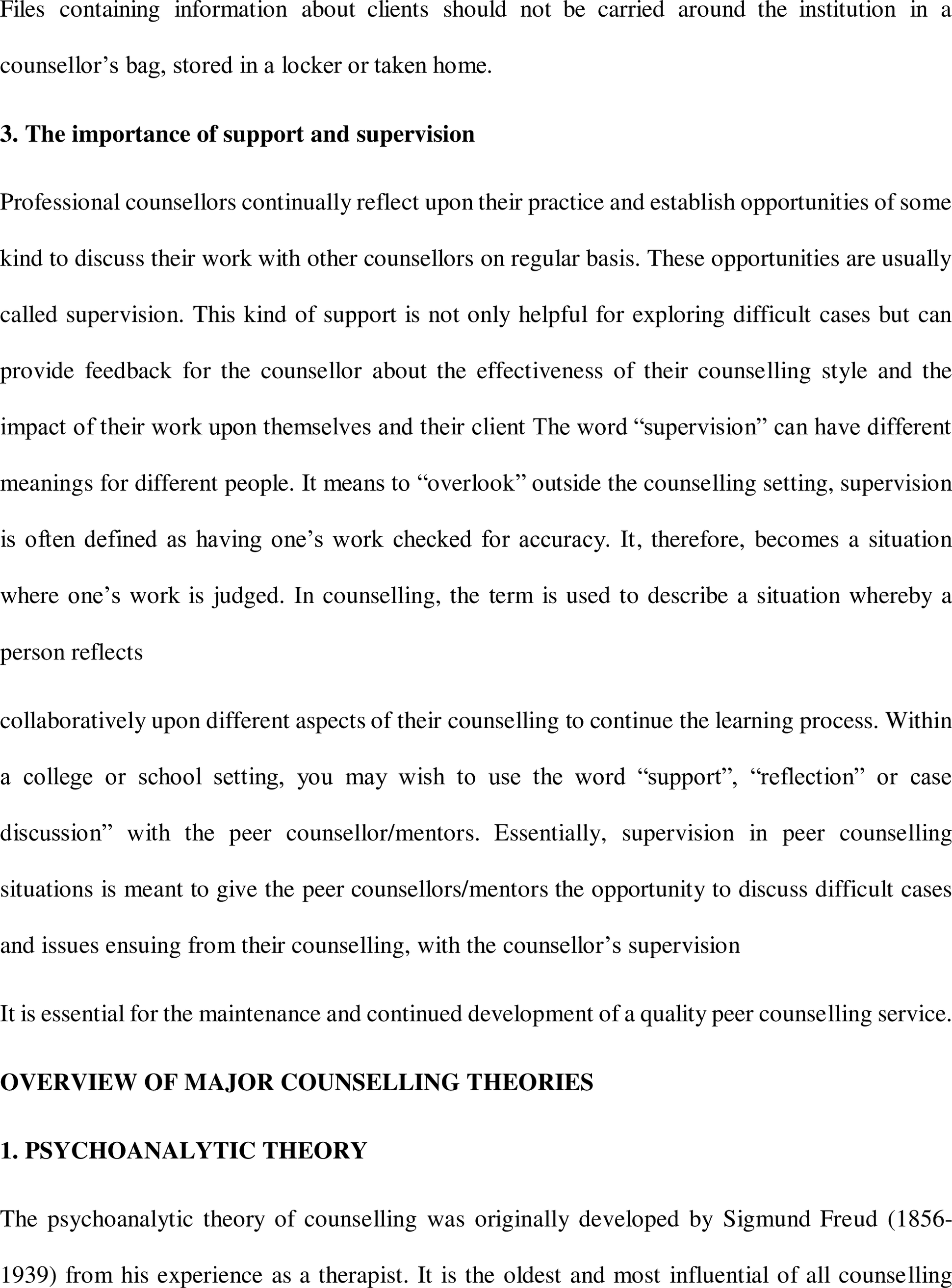

Supplement: S2 File — (ZIP) [file pdig.0000177.s002.zip › PACE Corrected/S2_PEER MENTORING-CURRICULUM.tif]

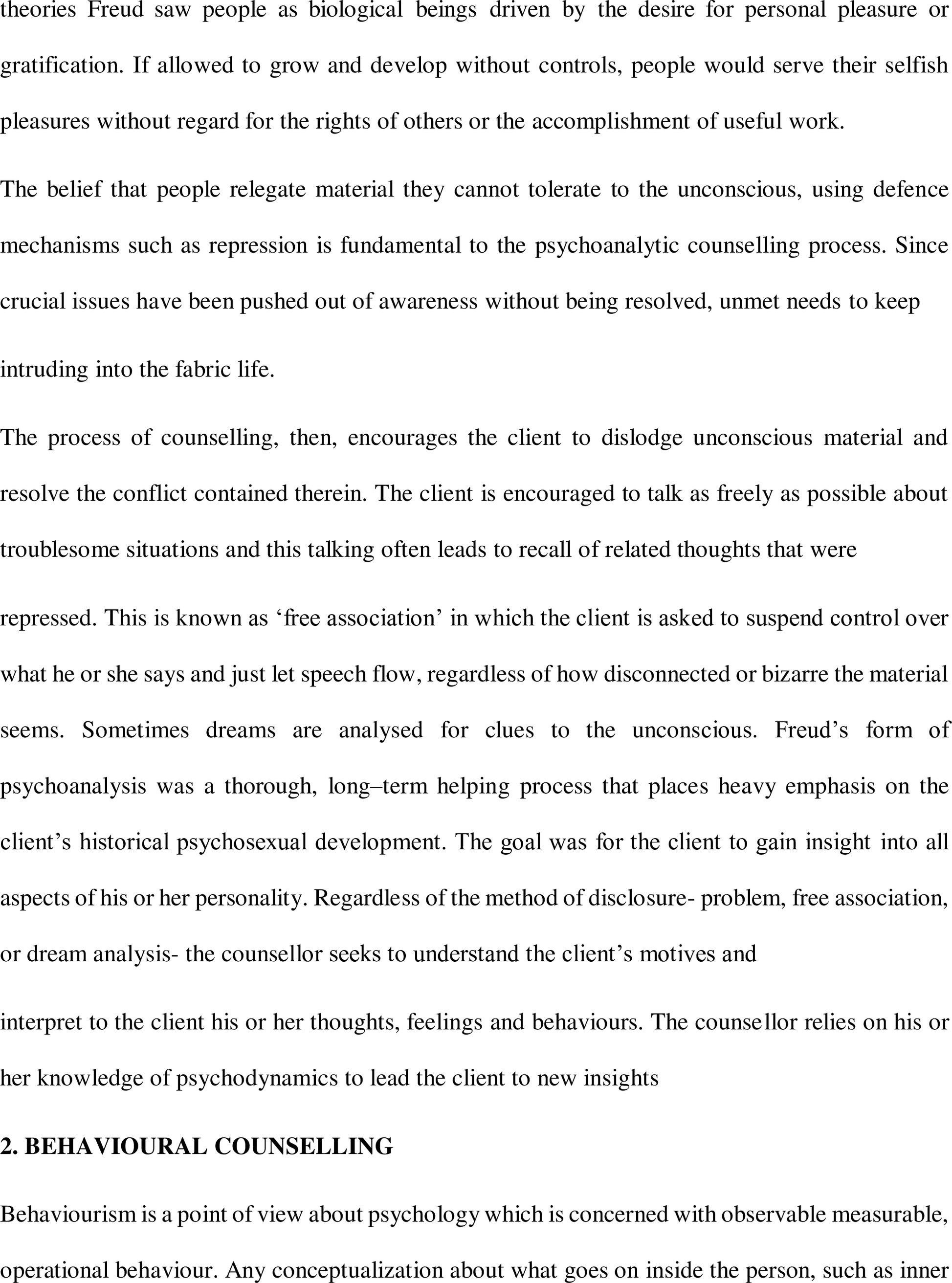

Supplement: S2 File — (ZIP) [file pdig.0000177.s002.zip › PACE Corrected/S2_PEER MENTORING-CURRICULUM.tif]

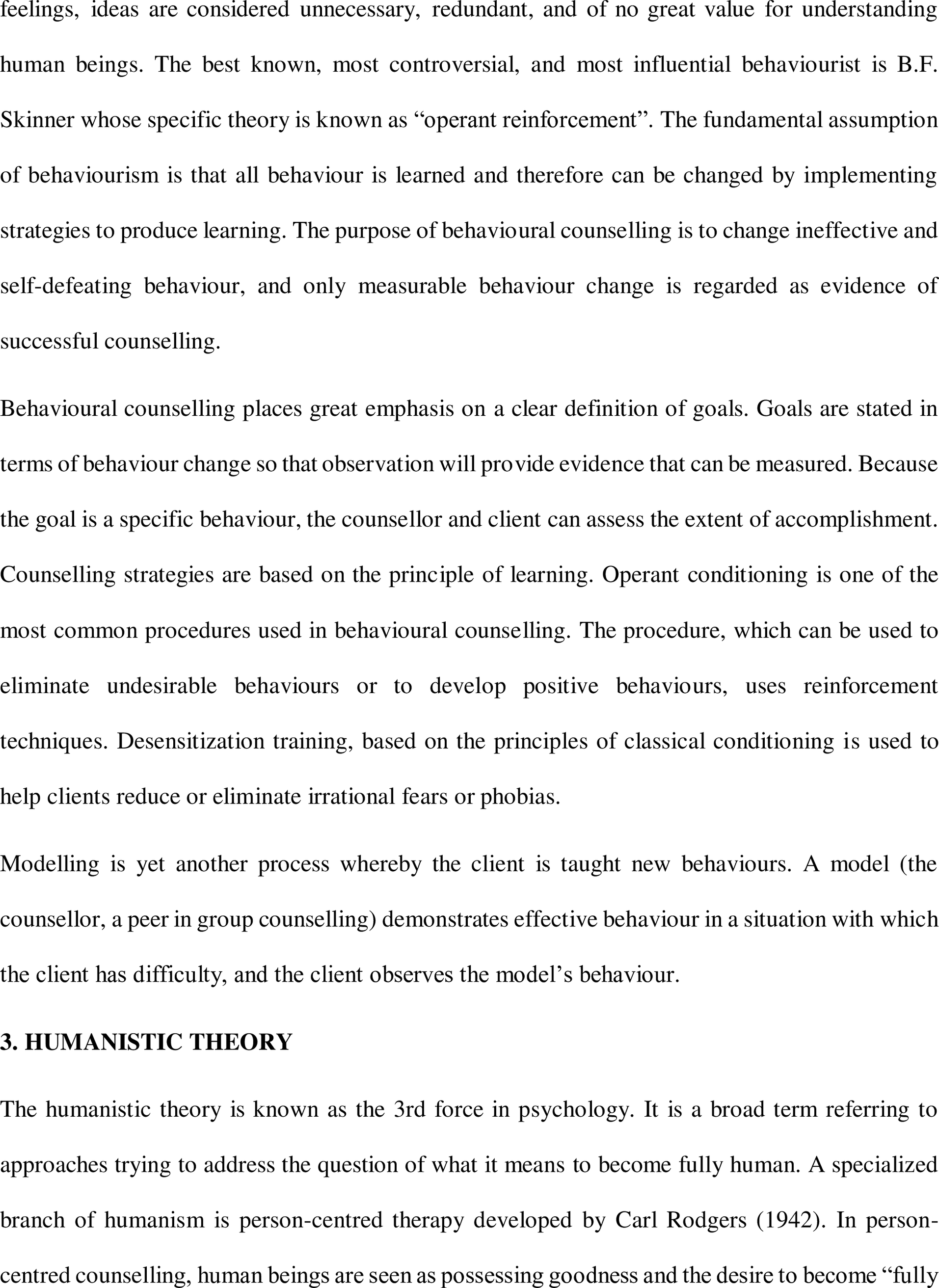

Supplement: S2 File — (ZIP) [file pdig.0000177.s002.zip › PACE Corrected/S2_PEER MENTORING-CURRICULUM.tif]

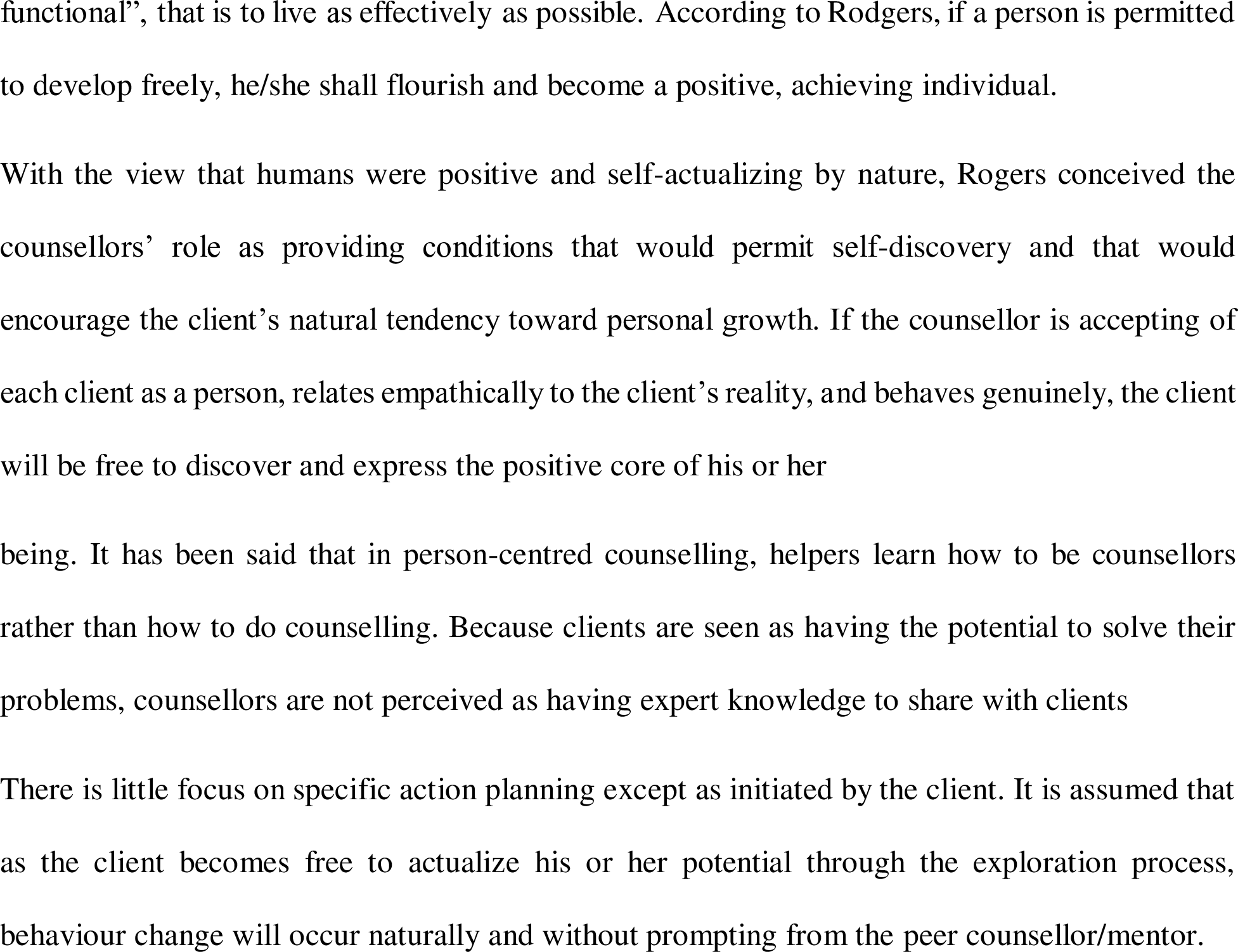

Supplement: S2 File — (ZIP) [file pdig.0000177.s002.zip › PACE Corrected/S2_PEER MENTORING-CURRICULUM.tif]

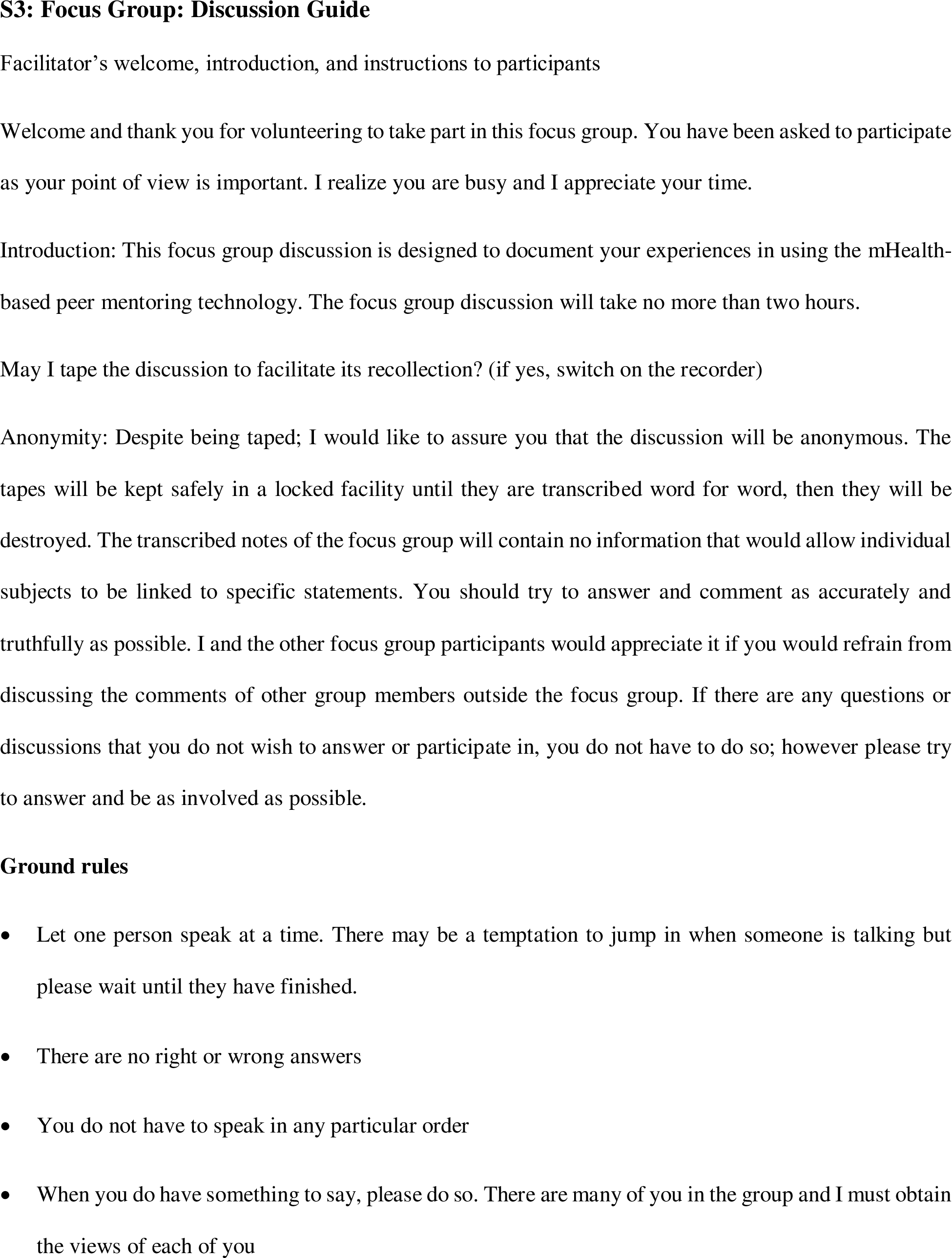

Supplement: S3 File — (ZIP) [file pdig.0000177.s003.zip › PACE Corrected/S3_Focussed Group Discussion Guide.tif]

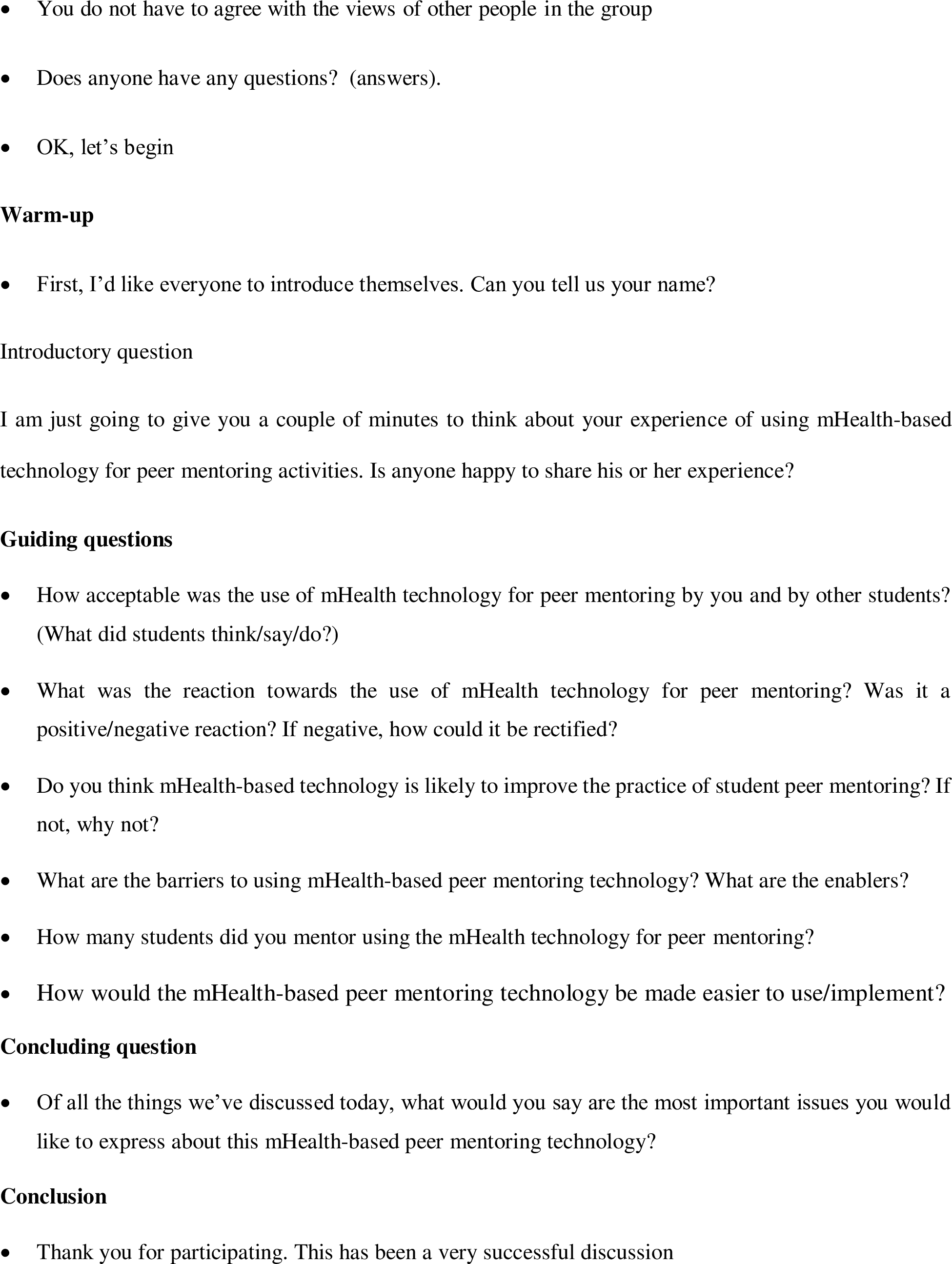

Supplement: S3 File — (ZIP) [file pdig.0000177.s003.zip › PACE Corrected/S3_Focussed Group Discussion Guide.tif]

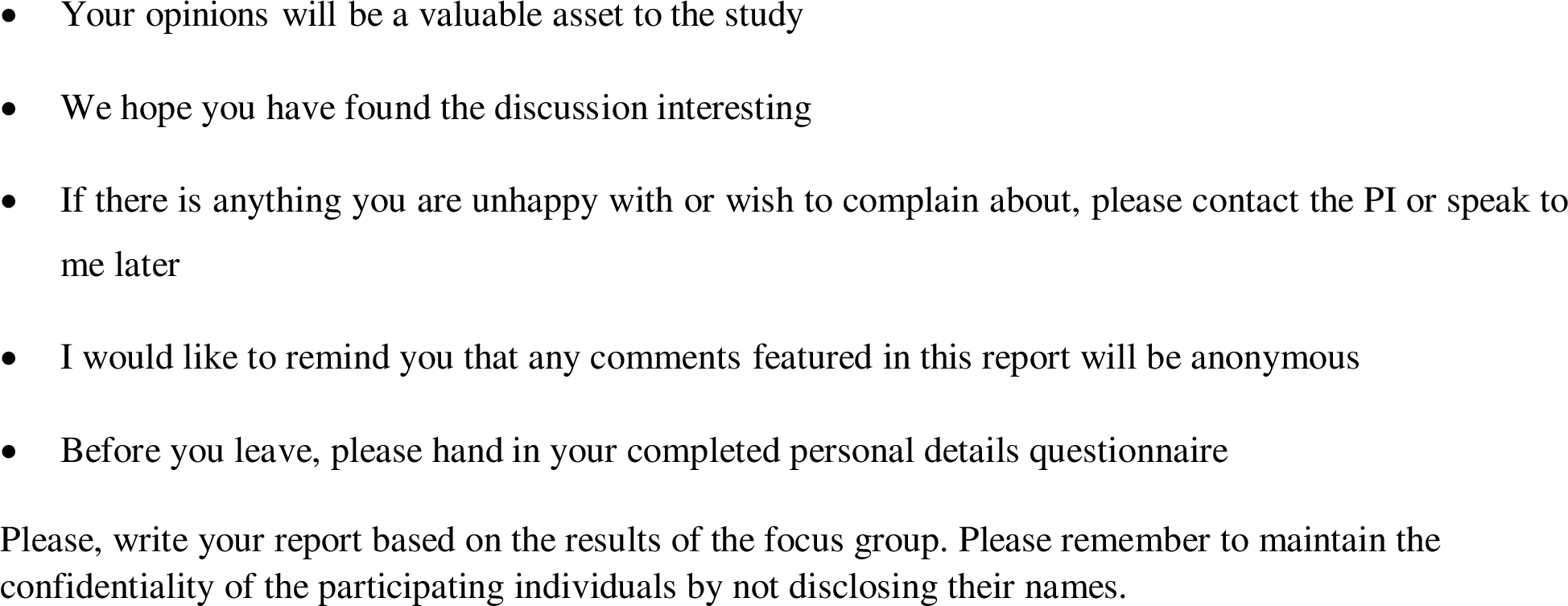

Supplement: S3 File — (ZIP) [file pdig.0000177.s003.zip › PACE Corrected/S3_Focussed Group Discussion Guide.tif]

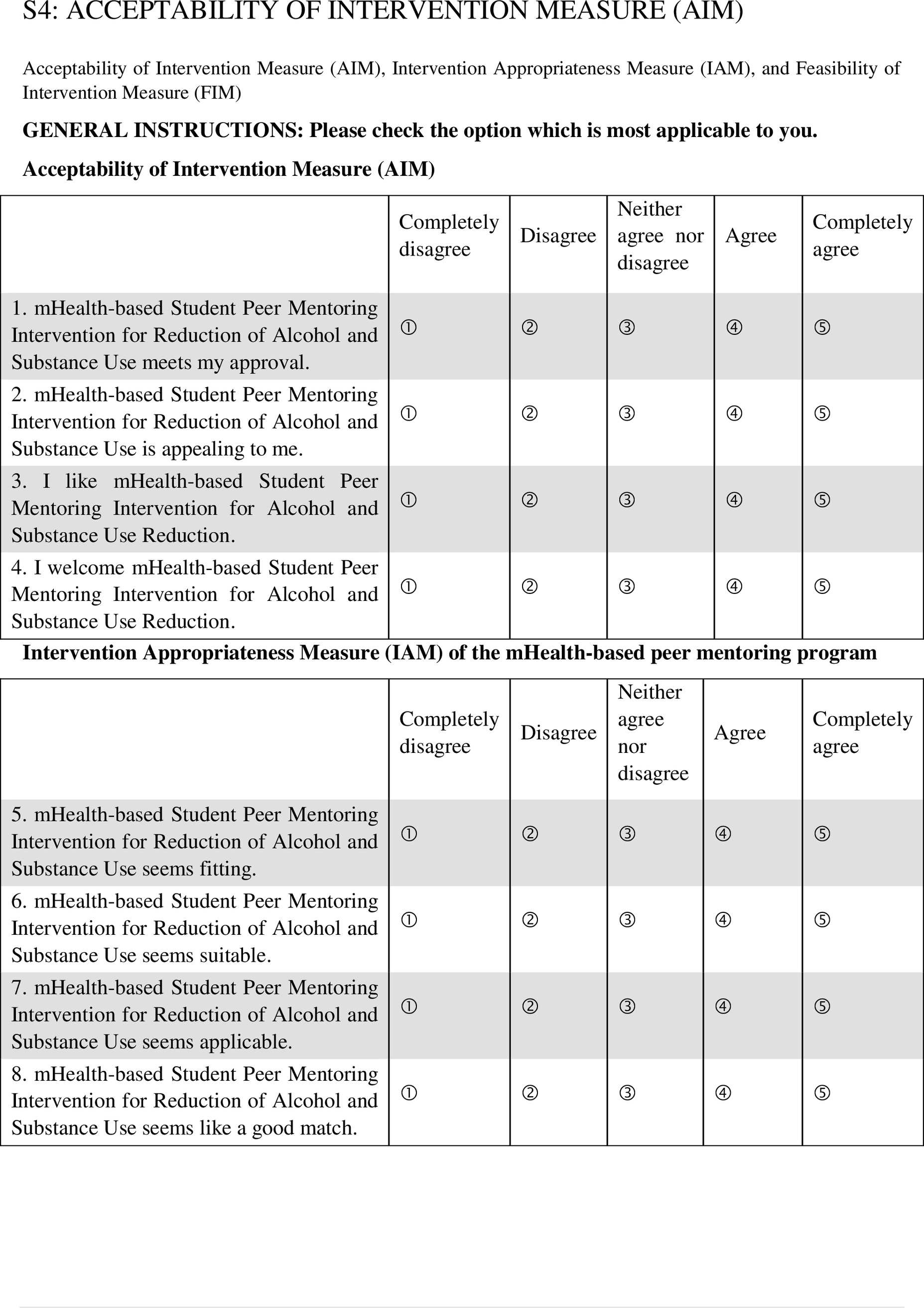

Supplement: S4 File — (ZIP) [file pdig.0000177.s004.zip › PACE Corrected/S4_Acceptabilty_TOOL.tif]

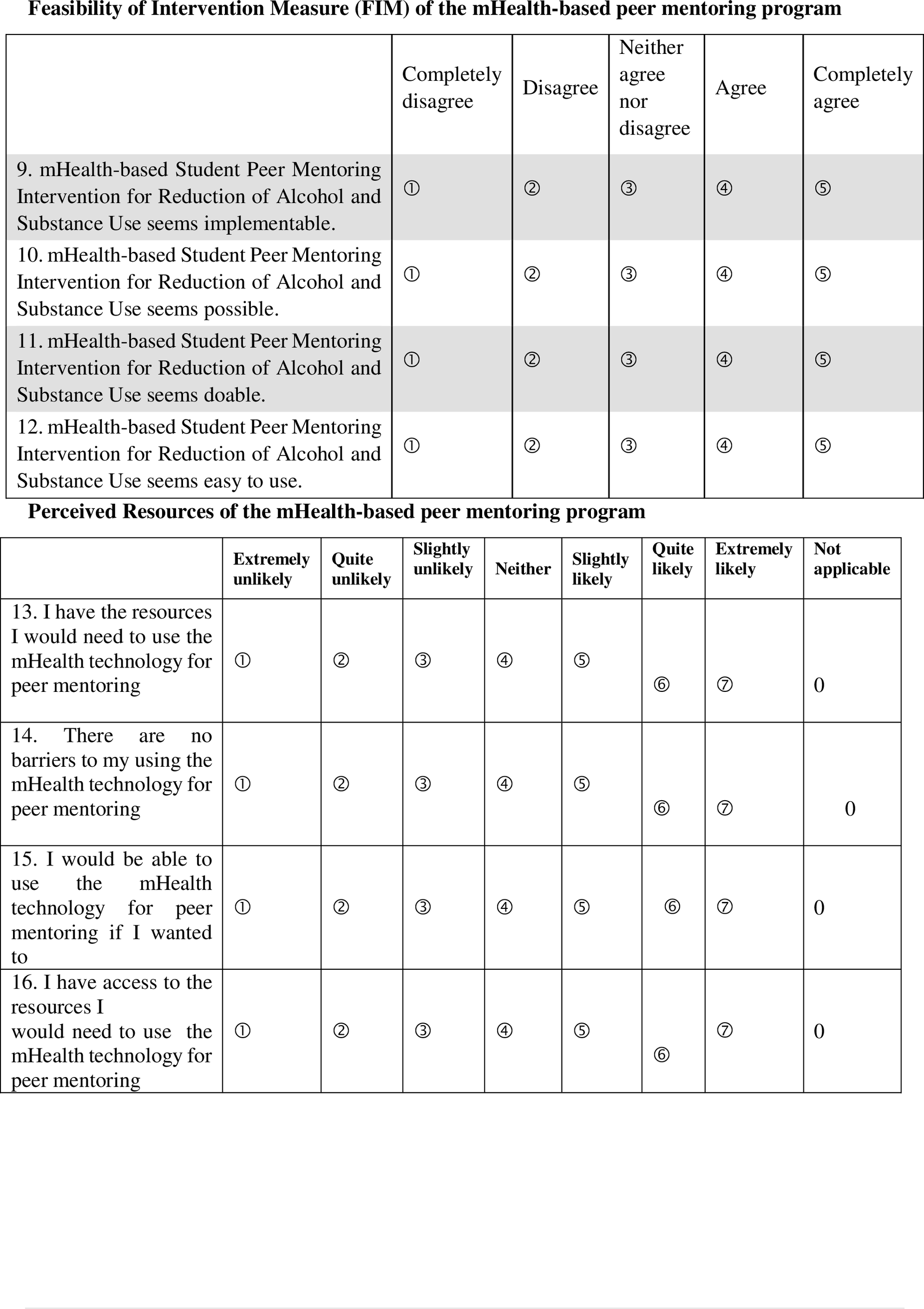

Supplement: S4 File — (ZIP) [file pdig.0000177.s004.zip › PACE Corrected/S4_Acceptabilty_TOOL.tif]

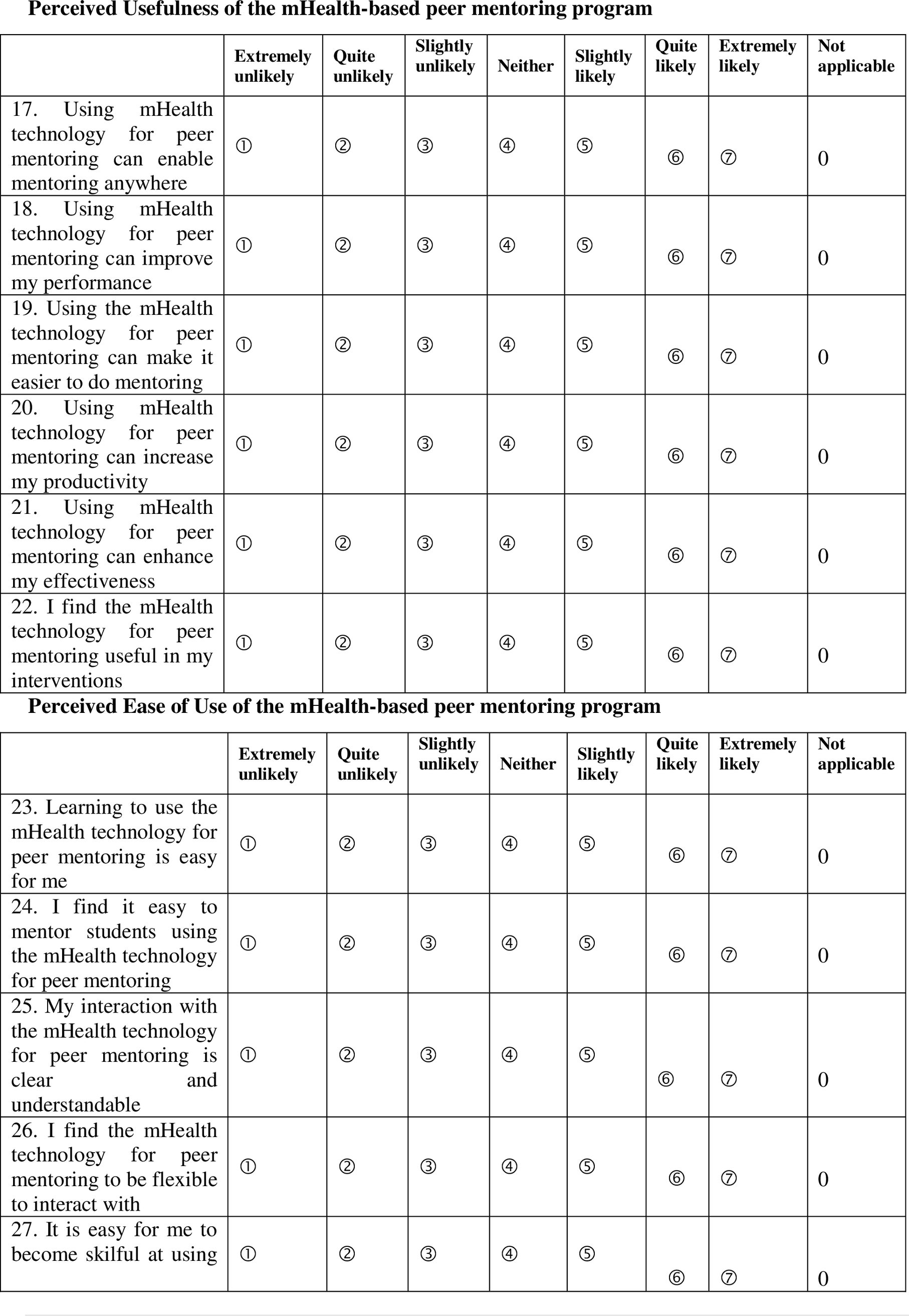

Supplement: S4 File — (ZIP) [file pdig.0000177.s004.zip › PACE Corrected/S4_Acceptabilty_TOOL.tif]

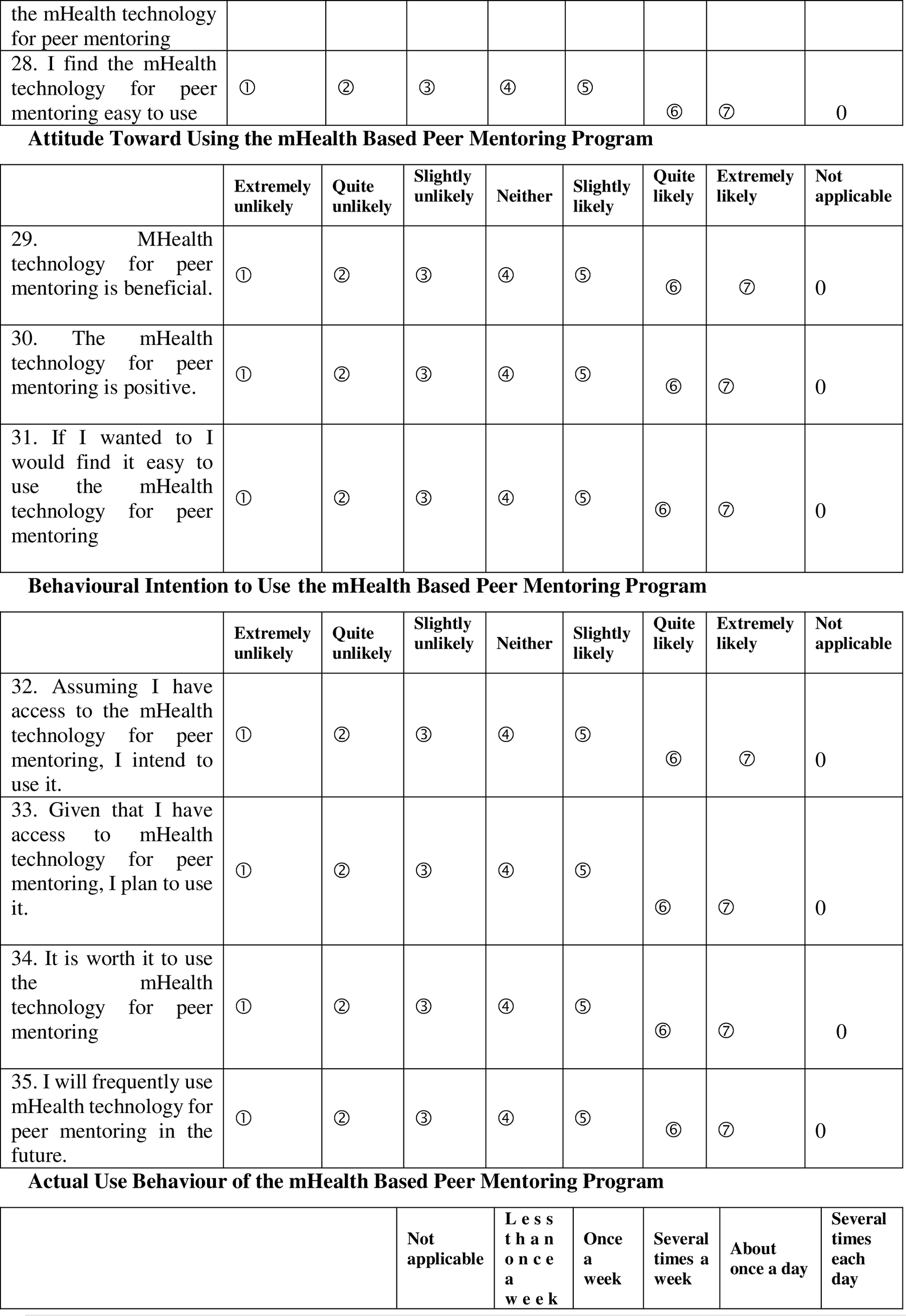

Supplement: S4 File — (ZIP) [file pdig.0000177.s004.zip › PACE Corrected/S4_Acceptabilty_TOOL.tif]

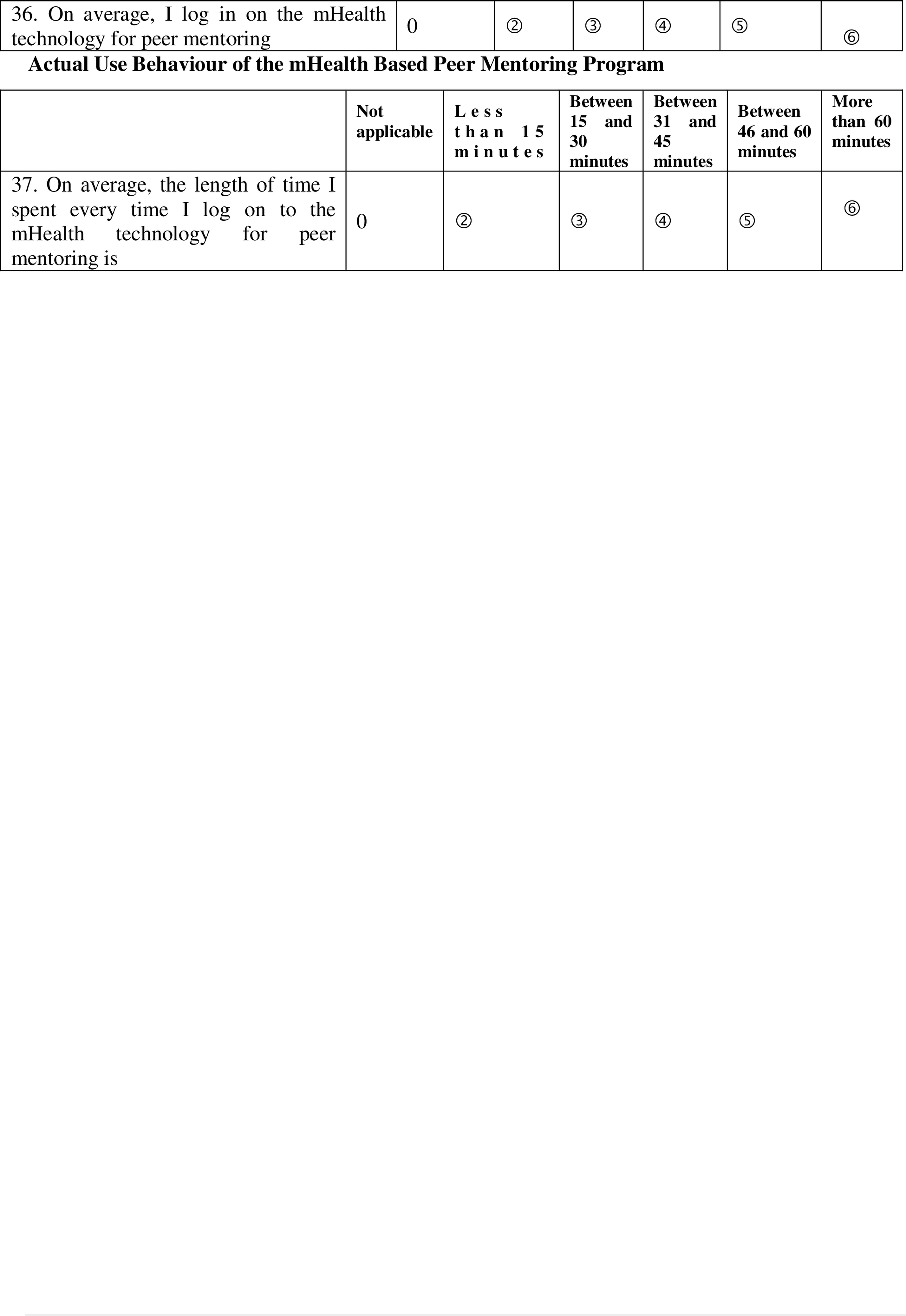

Supplement: S4 File — (ZIP) [file pdig.0000177.s004.zip › PACE Corrected/S4_Acceptabilty_TOOL.tif]
